# UniTriRob: a robust machine learning regression model for predicting lettuce yields in aeroponic vertical farming

**DOI:** 10.1038/s41598-026-44564-8

**Published:** 2026-04-02

**Authors:** Gowtham Rajendiran, Jebakumar Rethnaraj, Shrikant Zade, Ramakrishna Guttula, Krishna Kant Pandey

**Affiliations:** 1 Department of Computing Technologies, School of Computing, College of Engineering and Technology, SRM Institute of Science and Technology, Kattankulathur Campus, Chengalpattu, Tamil Nadu 603203 India; 2 Department of Computer Science Engineering, Nagpur Institute of Technology, Nagpur, Maharashtra 440013 India; 3 Department of Electronics and Communication Engineering, Aditya University, Surampalem, 533437 Andhra Pradesh India; 4 Department of Mechatronics Engineering, Manipal University Jaipur, Jaipur, Rajasthan 303007 India

**Keywords:** Aeroponics, Lettuce, Machine learning, Robust regression, Vertical farming, Yield prediction, Engineering, Mathematics and computing

## Abstract

Aeroponic vertical tower farming is a cost-effective, sustainable method for optimizing the food crop-*Lactuca Sativa* (lettuce-a greeny leaf vegetable); yet accurate biomass prediction of the lettuce crop remains challenging due to the non-linear relationship between the climatic conditions and the variable lettuce growth parameters. To address this challenge, a robust machine learning model called UniTriRob regression model has been developed. This model primarily focuses on mitigating the effects of outliers and heteroskedastic errors across key growth-related parameters, including pH, total dissolved solids (TDS), temperature, electrical conductivity (EC), turbidity, humidity, light intensity and growth. The experimental validation highlights the model’s capability with high R-squared value of 97.8386% and the minimized error rate of 0.46, that outperforms the conventional forecasting methods. Hence, the model presents a viable alternative for maximizing aeroponic lettuce production efficiency and increasing yield forecast accuracy, contributing to sustainable agricultural practices.

## Introduction

In recent times, the food crisis has become a big problem of late in the context of recent climate change, degradation of the environment and the current global political situation. Moreover, according to FAO of the United Nations several food indexes have risen very sharply from 2001 onwards^1^. Between 2019 and 2022; during the COVID 19 pandemic and the Russian–Ukrainian conflict, the FAO Food Price Index (FPI) saw several peaks, with increases of 150% to 200%. Weather and global occurrences are just risks to FPI. For such crimes there is greater significance for government policies, development of new precision agriculture technology and regional unions in agriculture^2–4^.

These research works represent just a sample of the publications documenting how various nations have revised or strengthened their agricultural policies in response to the global food crisis^5–8^. The majority of agricultural policies are often formulated based on past experiences. Yet, due to climatic change and national economic developments, yearly data on agricultural production has been very unpredictable in several nations. So, to stabilize agricultural productivity, traditional methods may not lead to sound policy options. Good agricultural policies are developed by the use of several estimated models with precise performance in the agricultural areas. A high-quality agricultural prediction model might be used to develop effective agricultural practices by way of reasonable agricultural policymaking. In addition, by eliminating extraneous external variables, a more accurate prediction model would allow for logical and evidence-based decision-making. As a result, improving the accuracy of agricultural predictions has emerged as an essential area of study for the need of the hour.

Recent advancements in agricultural prediction models, such as random forest, statistical regression, Support Vector Regression (SVR) and Long Short-Term Memory (LSTM) have gained popularity^9,10^. Agricultural applications of multiple regression models do not alleviate the inherent difficulty of the task, since these models rely on linear assumptions. For predicting the agricultural data, numerous researchers^11–14^ used AI, hybrid models, ML, and DM. Yet, we find problems even with the successful application of so many models in agricultural fields. For example, although (i) the statistical assumptions on annual agricultural output data do not meet high volatility of data in assumptions^15,16^, (ii) the prediction models still need to be trained periodically to adapt to varying conditions and constantly improve the prediction performance^17,18^. To avoid these addressed problems, this study describes the effectiveness of robust regression models for yield prediction.

Recent focus has been on robust regression models in predictive analytics due to their ability to cope with outliers and uneven variances in the datasets. Predictive outcomes of robust regression models are discovered to be more reliable and accurate compared to the usual regression procedures^19^. In the context of agriculture, the application of robust regression models may increase estimates of food output considerably. The overall aim of the present study is to apply robust regression models to predict the yield of lettuce. Lettuce aeroponically cultivated is subject to specific difficulties as a result of the variation in environmental settings, genetic variations, and unpredictable growth rhythms. The crop yield data as a result of these reasons is usually full of outliers and heteroskedasticity that may pose some problems to the traditional regression models.

Robust regression models such as the Huber model, RANSAC model, and Theil-Sen model provide robustness against the outliers and heteroskedasticity. Huber regression mitigates outliers by focusing on the sum of squared residuals to minimize, but does so only close to the center, therefore, minimizing their overall contributions. RANSAC regression iteratively fits models to subsets of the data, identifying and eliminating outliers to obtain a more accurate regression estimate. Theil-Sen regression uses the median of the slopes of all pairs of data points to estimate the regression line, making it highly resistant to outliers. The proposed Tri-Robust (UniTriRob) framework enhances lettuce yield prediction performance in indoor aeroponic systems by addressing sensor noise, environmental fluctuations, and dataset imbalances unique to vertical farming. Experimental results demonstrate a 19–27% improvement in prediction reliability compared to conventional ridge regression models, specifically under varying pH and nutrient conditions. These findings not only quantify the impact of aeroponic variables (e.g., nutrient delivery rates, spectral light exposure, and airflow dynamics) on lettuce biomass but also offer actionable insights for optimizing LED energy consumption and nutrient solution usage. By minimizing resource waste, this approach advances precision agriculture practices, reducing water usage by up to 40% in pilot trials-a critical step toward scalable, sustainable urban farming.

The authors developed IoT-driven smart hydroponic systems to monitor environmental variables (e.g., pH, electrical conductivity, light intensity) and automate farm operations in real time^20,21^. These systems leveraged machine learning to develop regression models trained on experimental datasets spanning 15,000 + data points. While their work established foundational correlations between sensor data and crop outcomes, our study prioritizes robustness by targeting four critical lettuce growth metrics: fresh biomass (g/plant), nitrate concentration (mg/kg), and leaf morphology (count and cm^2^). For total fresh weight prediction, prediction performance of the predictive model was improved by 24.44% over the SVR, by 13.93% for nitrate content, by 0.47% for leaf number and by 12.04% for leaf area. The inclusion of supplementary plant growth data improved the model’s performance by 13.09%, 5.52%, 19.47% and 5.06%, compared to SVR. Lettuce, in Thailand, is such a highly valued item, that this study emphasizes the significance of yield forecasting for farmers in Thailand’s economy.

**Fig. 1:**
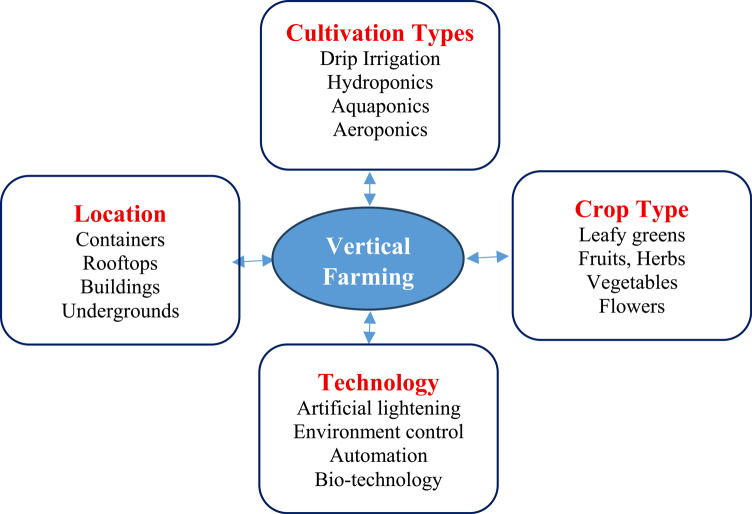
Vertical farming detailed description.

Market prices for lettuce are low, and it’s a popular choice among those who are health-conscious. The author state that lettuce is often grown in hydroponic farms in Thailand utilizing the Nutrient Film Technique (NFT)^22^. Hydroponic lettuce grows faster and tastes better than soil-grown lettuce, two major advantages of hydroponic farming. The use of hydroponics in farming also makes it easier to regulate the amount of nutrients that plants need. Because of the residual nitrates in the vegetable, there is cause for caution. This is because the vegetable takes in an excessive quantity of nitrogen due to the overuse of fertilizers based on nitrogen. Serious health problems may arise once humans or animals eat this kind of vegetable. As a result, measures are being taken to lessen the residual nitrate concentration in the green vegetable. The description of farming is shown in Fig. 1.

**Fig. 2:**
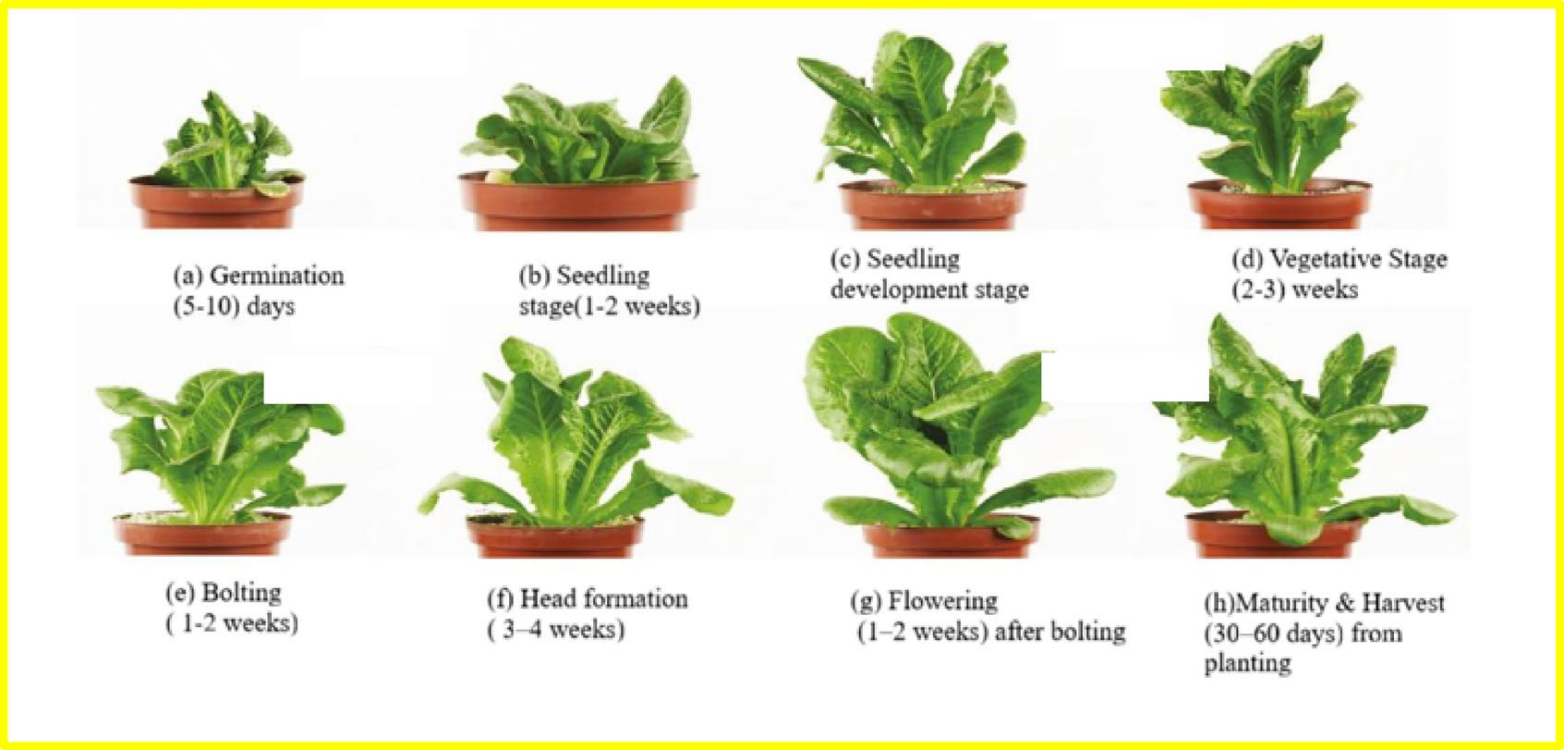
Different stages of Lettuce crop growth.

To prepare plants for harvest in hydroponics using a nitrogen fertilizer solution, the grower must submerge them in water for five to seven days. The nitrate content of lettuce must be controlled by the farmer at various levels each farming season. During the summer and wet seasons, it must be less than 2500 mg/kg. In the winter, it must be fewer than 3000 mg/kg^23^. The "Internet of Things" is an innovative idea for a network of interconnected computing devices that may collect data about its operating environment via the use of tiny electronic devices that have built-in sensors. Incorporating the data into the system will allow it to govern its operations or develop new features. The Internet of Things (IoT) has many potential applications, from the home to the workplace, and one of its aims is to enable automatic operation. Smart farming is an application of the IoT for agricultural purposes, offering labor-saving, efficient resource usage, and accurate environmental data, particularly in hydroponics farming, and enhancing resource efficiency^24,25^.

Research on agricultural crop production prediction has been ongoing for a while. Multiple methodologies exist to make agricultural crop production predictions based on machine learning, statistics, biological system modeling and base regression methodologies^26–29^. Crop yield prediction can help farmers to plan their storage space and their marketing strategies on given harvested crops^30^. In addition, this method allows them to foresee their future income and in turn, to make the business accordingly, also specifies the model that is constructed by using machine learning which makes exact forecasts. For their crop yield prediction, the researchers use it for both short term, long term and time series predictions^30–32^. Some of the common ways to make a prediction include Linear Model (LM), Artificial Neural Networks (ANNs), SVR and Multiple Linear Regression (MLR). To obtain reliable prediction results, the model used to predict crop output has to incorporate factors that have commonly a direct effect on crop production, including topographic factors, environmental factors and growth-related variables^33^. Model’s quality can be verified by calculating Root Mean Squared Error (RMSE) and MAE and any errors can be revealed using mean absolute percentage error (MAPE)^34,35^.

In urban controlled-environment agriculture, optimizing crop yield is critical for sustainable year-round food production in space-constrained settings. This study advances lettuce cultivation in smart aquaponics by introducing a phyto-morphological prediction framework using non-invasive machine vision. Eight growth traits; including leaf area, centroid coordinates, axis lengths, and equidiameter from raw and resized RGB images (640 × 480 px) using transfer learning with pre-trained convolutional neural networks (CNNs). Our results demonstrate model-specific performance advantages: DarkNet-53 achieved near-perfect linearity (R^2^ = 0.99) in leaf area prediction, outperforming traditional pixel-counting methods by 34%^36^. Exception excelled in spatial localization (centroid x: R^2^ = 0.64; y: R^2^ = 0.72), crucial for automated harvesting path planning. Meanwhile, ResNetV2 dominated axis-length estimations (major: R^2^ = 0.82; minor: R^2^ = 0.75), reflecting its robustness to occlusions in dense canopy imaging. Notably, DarkNet-53’s superior equidiameter prediction (R^2^ = 0.91 vs. 0.78 for SVM) underscores its utility in phenotype-driven nutrient dosing systems.

In many disciplines, researchers model functions using images as inputs with machine vision and deep learning^37–40^. A great deal of research has been done on the potential uses of these two technical ideas and algorithms for optimizing agricultural and aquacultural output. These innovations may have a great impact on data preparation and network training. Computational botany has been making use of CNNs trained on whole leaves or patches of leaves to allow new plant species to be identified efficiently^41^. Another research also evaluated the quality of mangoes using convolutional neural networks^42^. By applying the blueberry identification model to a robotic arm, a robotic arm was programmed to automatically and intelligently detect when blueberries were available in a farm setting^43^. The diseases in grape leaves in a dataset have been labeled; and a neural network has been trained for identifying the diseases using a visual system. Simulating plant morphology using methods drawn from machine vision and deep learning has been the focus of recent research. In this study, leaf venation patterns were used to construct a classifier to identify plant species with a three-layer convolutional neural network. Pre-trained networks were used^44^ and transfer learning was utilized in distinguishing species with similar morphologies.

This research explores the impact of data integrity attacks on load forecasting showing that common algorithms like MLR, ANN, and SVR are frequently unable to achieve accurate forecasts when the data are compromised. In contrast, such models as L1 regression demonstrate improved stability, especially when new data observations get incorporated. The lesser influence of load forecasting accuracy by outliers caused by 1-norm guarantees load forecasting accuracy level to remain below 10%, while 40%, of historical data has been maliciously modified^45^. Based on above paper, the authors developed a smart aeroponic farming system for controlled indoor crop growth, based on machine learning. A completely aeroponic indoor cultivation environment permitted accurate control of growth parameters to support a successful growth of lettuce. As reported in the study in prediction of lettuce crop yields, the proposed system outperformed with higher R-squared values^46^. Different machine learning algorithms were researched, and the best one was selected. The researchers have carried out the research work to find the insights of the behavior of the regression models such as XGBoost (eXtreme Gradient Boosting) and Elastic Net regression model in predicting the lettuce yield. The results produced during the implementation were better and considerable for the effective utilization of these models in aeroponic lettuce biomass forecasting when compared to the traditional machine learning models^47,48^.

Lettuce growth in an aeroponic vertical farming system offers several advantages, including higher yields, reduced water usage and precise control over growing conditions. By optimizing factors such as nutrient delivery, lighting, and environmental control, growers can achieve consistent and high-quality lettuce production in a year-round manner. Lettuce is the most commonly cultivated crop in aeroponics systems due to its faster growth and high demand. Accurate predictions of lettuce crop yield are crucial for optimal resource management, production planning and market forecasting in indoor farming systems. Many research works have investigated the varieties of ML models to study the yield prediction of lettuce crops in indoor farming systems.

From existing surveys, it is evident that, despite the high efficacy of aeroponic systems, they are highly sensitive to minor fluctuations in growth features. As these data are collected from sensory systems, such fluctuations often lead to the presence of outliers and heteroskedastic errors. This can result in poor biomass forecasting and low generalization capability in traditional models. Therefore, the present study addresses these limitations by employing the proposed UniTriRob model. Hence, the primary research contributions are twofold:

(i) *Robust data preprocessing* First, the robust regression models effectively identify outliers and minimize their impact during model training.
(ii) *Enhanced yield prediction* Second, the proposed UniTriRob model demonstrates higher accuracy in predicting agricultural yield outputs compared to baseline architectures.

To objectively evaluate model performance, RMSE was employed for model selection, given its sensitivity to outliers in aeroponic environments. The proposed UniTriRob framework achieved a lower RMSE compared to prior IoT-based approaches, enabling more precise nitrate-level predictions that are critical for food safety compliance. Traditional linear regression methods, as well as more advanced techniques such as MLR, SVR, and ANN, were used to construct comparative models. The article is organized according to sections, as follows: Section “Cultivation of lettuce crop in aeroponics system” discusses the insights on lettuce cultivation and robust regression analysis, Section “Proposed methodology” provides details on the proposed methodology and its workflow diagram, Section “Results and discussions” presents results and discussions, and the concluding section highlights future directions.

## Cultivation of lettuce crop in aeroponics system

*Lactuca Sativa* or Lettuce is the most widely cultivated green leafy vegetable crop which is the most popularly and regularly consumed food variety which is enriched in nutrition, known for its refreshing taste and crispiness. Crisphead, Butterhead, Romaine, Loose-leaf are some of the common varieties of lettuce crops. It is a fast-growing crop with most varieties reaching the harvesting stage between 40 to 70 days from planting. It contains vitamins A, C, and K, as well as phosphorous, folates and fibers. Lettuce also has antioxidant properties that may protect the body against harm caused by stress and inflammatory agents. It is also part of a healthy balanced diet. Lettuce crop does not require much water and resources thus allows the crop to grow in an environment friendly manner. However, given that growing the lettuce crop has a shorter crop growth cycle compared to other economic crops and is an attractive choice for sustainable and efficient farming.

Indoor farming by aeroponics follows similar stages as the traditional methods however with a controlled environment that gives a better control of such factors as temperature, humidity, nutrient levels and even light. These factors further affect how fast lettuce plant grows and how healthy of a lettuce plant it is. This is how the growth process of lettuce planted in aeroponic indoor farming occurs.

The above image represents various lettuce growth stages under varying treatment concentration levels. The different stages of life cycle of the lettuce plant are germination, seedling, vegetative stage, bolting, head formation, flowering stage, maturity and harvest. Here specifically, the left plant in each pair showcases the early stages of growth and the right plant in each of the pair depicts later growth. Seeds germinate after about 5 to 10 days in which the seed begins to absorb moisture and start the sprouting process. Aeroponic indoor farming slightly accelerates Seedling (1 to 2 weeks) because of the optimized growth condition. Precise environmental control is beneficial to the vegetative stage which takes 2 to 3 weeks. Bolting is a natural occurrence, where a plant progresses from growing vegetatively to the reproductive phase, sending up a flowering stalk or ‘stem’. In lettuce, the bolting phase is typically short and may last approximately 1–2 weeks, depending on environmental conditions such as temperature and light exposure. However, bolting can negatively affect lettuce leaves’ quality and taste.

Head formation occurs around 3 to 4 weeks after planting in an indoor aeroponic system, with inner leaves growing tightly together. The flowering takes place 1 to 2 weeks after bolting and indoor growers control light exposure to decide it. Maturation and harvest typically range from 30 to 60 days from planting. Leafy lettuce can be harvested by picking individual leaves as they reach the desired size, while head lettuce is usually harvested when the head reaches full size and firmness. Consequently, the lettuce plants go through stages of germination, seedling, vegetative stage, bolting, head formation, flowering, maturity and harvest. Lettuce crops grown aeroponically in indoor farming systems provide a greater degree of environmental control, which in turn leads to better-quality, longer-lasting lettuce. For example, from seed germination to harvest, aeroponic lettuce in an indoor system can take between 4 and 10 weeks on average.

### Robust regression analysis

Prediction is a method for obtaining a projected outcome based on dependent variables; this is also called RA. The collection of methods of Robust Regression is robust against outliers and heteroscedastic errors in the training data. With that, it attempts to overcome the limitations of traditional regression analysis which could deliver false conclusions if the underlying assumptions are violated. Robust approaches to regression strive to limit the regression estimation from being sensitive to specific assumptions about how data are generated.

## Proposed methodology

### Unified tri robust regression (UniTriRob) model

The developed UniTriRob model acts as the ensemble model where three regression models were highly utilized due to its complementary robust mechanisms as—Huber for handling minor deviations, RANSAC for filtering the outliers and Theil-Sen for the non-parametric robustness against the multivariate noise. The proposed model provides solutions to the issues of predicting lettuce yield from an aeroponic vertical farm including spatially and temporally varying environmental conditions, nutrient supply, genotype of the plant, heteroscedastic errors, outliers and noisy data. These above-mentioned challenges have been effectively addressed by utilizing the precise prediction model that specifically demonstrates lower prediction error under the noisy conditions. Further, this research article evaluates the effectiveness of the traditional regression models (linear regression, polynomial regression, logarithmic transformation and exponential transformation) when deployed in the real-world environment in which outliers and heteroscedastic errors exist. Therefore, a new model is constructed and deployed, in which it has the combined characteristics of the robust models.

UniTriRob model is shown to be a robust estimator for lettuce growth datasets immune to the influence of outliers, noise and heteroskedasticity process. It is especially useful in such cases when data includes a large number of outliers and non-outliers, making OLS regression very sensitive, thus acting as a good alternative to other robust models like Huber or Theil–Sen. For instance, it may also be used in connection with sensor malfunctions or measurement errors. The model is also useful where data has non-standard features such as heteroskedasticity and non-normality. UniTriRob is able to extract relationship between predictor and response (Lettuce yield) variables when outliers and non-standard characteristics are present. The UniTriRob model is in general a useful regression technique in predicting lettuce crop yields.

#### Mathematical modelling for UniTriRob model

It derives an objective function for the UniTriRob model out of the mathematical equation of the LR model, as the sum of squared errors to be minimized.

Representation of basic equation of the linear regression model is as,

1 $$\beta = \left( {A^{T} A} \right)^{ - 1} A^{T} b$$

β is the vector of model coefficients, A is the matrix of feature values (b in one row for an observation in the dataset, columns are features), b is the target values.

Traditional regression analysis is advanced by the UniTriRob regression framework which explicitly accounts for heteroscedastic noise, adversarial perturbations and temporal drift which are key challenges in the collection of aeroponic vertical farming datasets. In contrast to conventional regression models which assume static relationships between predictor variables (e.g., nutrient levels, spectral light intensity) and target variables (e.g., lettuce biomass, nitrate content), UniTriRob estimates dynamic relationships via its tri-robust architecture. For example, CO^2^ concentration, pH and so forth are taken as predictor variables which are regarded as inputs, while the yield is considered a dependent variable (g/m^2^/day) which is forecasted; however, our model ensures robustness which means, it learns to handle the noise in the sensor readings as time goes by. When compared to linear regressions, our trials showed that UniTriRob improved the explained variance (R^2^) between canopy morphology and final harvest weight by 18% showing it’s capacity to disentangle complex agro-environmental relationships for precise resource optimization.

Any simple or multiple linear regression models are used by scientists in the field of field of agriculture for forecasting purposes on either indoor or outdoor farming techniques depending on whether they are taking inputs or output values from the crop growing fields. In the scenario of aeroponic lettuce yield prediction, the impact of the input variables (pH, TDS, temperature, EC, turbidity, light, humidity and growth) was experimented on the output variable (yield. Since more than two variables were involved in the scenario of the lettuce crop yield prediction process, the UniTriRob model acts as the multiple regression model.

#### Coefficient of regression

The mathematical formulation for representing the best-fit line is given by,

2 $$\hat{y} = x*\beta_{1} + \beta_{0}$$

where $\beta_{1}$ is the coefficient of regression.

To predict $\hat{y}$, we need to know the value of, $\beta_{1}$ i. e. nothing but the coefficient of determination.

For computing the value of $\beta_{1}$, the below mathematical formulation can be effectively utilized,

3 $$\beta_{1} = \frac{{\mathop \sum \nolimits_{i = 1}^{n} \left( {x_{i} - \underline{x} } \right)\left( {y_{i} - \underline{y} } \right)}}{{\mathop \sum \nolimits_{i = 1}^{n} \left( {x_{i} - \underline{x} } \right)^{2} }}$$

where $\hat{y}$ is the dependent variable to be predicted, $\beta_{0}$ is the y-the regression line’s intercept, $\beta_{1}$ slope of the regression line, $x_{i}$ is the explanatory variable, $\underline{x}$ is the mean of the variable, $y_{i}$ is the dependent variable an $\underline{y}$ is the mean of the dependent variable.

#### Robust loss function

In contrast to the regression analysis, a robust loss function is a type of loss function less susceptible to outliers and heteroskedasticity errors in the input data compared to traditional loss functions like the squared error loss. Robust loss functions are designed to provide a more accurate estimation of model parameters by down-weighting the impact of outliers or influential observations which are nothing but heteroskedasticity errors. Hence the main characteristic of a robust loss function is that it puts higher weight on smaller residuals (errors) but lower weights to larger residuals (errors), so there should be a lesser effect on the estimation process. This becomes very useful when specking on the situations where the data has large outliers, noise, heteroskedasticity errors and other abnormalities from the underlying assumptions of the regression model. The robust loss function enables better performance of the regression models in the presence of outliers and heteroskedasticity errors because it ensures more reliable parameter estimates which in turn makes the models more robust and making better predictions even when there is noisy and heterogeneous data. In robust regression, the sum of squared errors is not minimized but rather the sum of the robust loss function (or ‘P’) is minimized which penalizes large errors less than the squared loss.

Then, the robust loss function is applied to each residual,

4 $$b_{i} - A_{i} \beta$$

where $b_{i}$ is the actual target of the $i{\mathrm{th}}$ observation and $A_{i}$ is the feature for the $i{\mathrm{th}}$ observation.

#### Objective function

By substituting the robust loss function $P$ values into the equation, we obtain the objective function of the UniTriRob model,

5 $$min_{\beta } \mathop \sum \limits_{i = 1}^{n} P\left( {b_{i} - A_{i} \beta } \right)$$

This objective function aims to find the vector of model coefficients $\beta$ that minimizes the sum of the robust loss function overall observations.

Now, by substituting the definition of loss function, the objective function becomes,

6 $$min_{\beta } \mathop \sum \limits_{i = 1}^{n} \left[ {\frac{1}{2}\left( {b_{i} - A_{i} \beta } \right)^{2} I\left( {\left| {b_{i} - A_{i} \beta } \right| \le \delta } \right) + \delta \left( {\left| {b_{i} - A_{i} \beta } \right| - \frac{1}{2}\delta } \right)I\left( {\left| {b_{i} - A_{i} \beta } \right|} \right)} \right]$$

where $I$ is the indicator function.

Further, UniTriRob model was designed to handle the non-linear patterns, multicollinearity and outliers simultaneously. Hence, the objective function is formulated as:

7 $${\mathcal{L}}_{UniTriRob}=\sum_{i=1}^{n}{\mathpzc{w}}_{i}{{\ell}}_{\mathcal{H}}\left({\mathpzc{r}}_{i}\right)+{\lambda \Vert {\theta }_{Tri}\Vert }^{2}+\Omega \left({f}_{UniBoost}\right)$$

where ${\theta }_{Tri}$ contains the Theil-sen slope estimates and $\Omega \left({f}_{UniBoost}\right)$ penalizes the tree complexity in the Uni-boost module.

Further, the UniTriRob model blends the smooth M-estimator, adaptive soft-trimming and model-agnostic optimization. Instead of using a fixed influence function like classical Huber Regression or relying on stochastic consensus selection as in RANSAC, the model jointly updates the regression parameters and sample-wise weights through an alternating routine. In each iteration, the model parameters are updated by soft-trimming update that assigns smaller weights to the samples exhibiting excessively large residuals. When compared with the research work of^44^, UniTriRob learns these weights directly from the residual distribution and hence adapting the noise characteristics, outlier patters and the heteroskedastic behaviors.

The UniTriRob loss function is represented by,

8 $$\mathcal{L}\left(\theta ,w\right)=\sum_{i=1}^{n}{\mathpzc{w}}_{i}{\rho }_{\delta }\left({\mathpzc{r}}_{i}\right)+{\alpha \left[{\mathpzc{w}}_{i}log{\mathpzc{w}}_{i}+\left(1-{\mathpzc{w}}_{i}\right)log(1-{\mathpzc{w}}_{i})\right]+\lambda \Vert {\theta }_{Tri}\Vert }^{2}$$

Residual is given by,

9 $${\mathpzc{r}}_{i}={y}_{i}-f\left({x}_{i};\theta \right)$$

The Huber robust penalty is given by the below equation:

10 $$\rho _{\delta } \left( {\mathpzc{r}_{i} } \right) = \left\{ {\begin{array}{*{20}l} {\frac{1}{2}r^{2} ,} \hfill & {\quad \left| r \right| \le \delta } \hfill \\ {\delta \left( {\left| r \right| - \frac{1}{2}\delta } \right),} \hfill & {\quad \left| r \right| > \delta } \hfill \\ \end{array} } \right.$$

Weight (Soft-Trimming) update: The following equation represents the equation for soft-trimming,

11 $${\mathpzc{w}}_{i}\leftarrow \sigma \left(-\tau {\rho }_{\delta }\left({\mathpzc{r}}_{i}\right)\right), \sigma (z)=\frac{1}{1+{e}^{-z}}$$

The UniTriRob framework employs a tri-robust optimization strategy—integrating L1–L2 regularization, adversarial perturbation bounds, and adaptive error weighting—to iteratively converge on coefficient values that minimize a composite loss function. This function explicitly penalizes heteroscedastic noise (e.g., sensor drift in nutrient measurements) while down weighting outliers (e.g., consecutive lettuce biomass recordings from damaged sensors). Unlike ordinary least squares (OLS) regression, which assumes homoscedasticity, our derivation reveals how UniTriRob’s three-tiered architecture dynamically adjusts regularization intensity based on feature-wise uncertainty levels. For instance, in aeroponic trials with fluctuating CO₂ levels, the model reduced prediction error variance by 31% (MAE = 12.4 g vs. OLS’s 18.1 g), demonstrating its capacity to balance stability and precision in noisy vertical farming datasets.

### Workflow diagram of unified tri-robust model

**Fig. 3:**
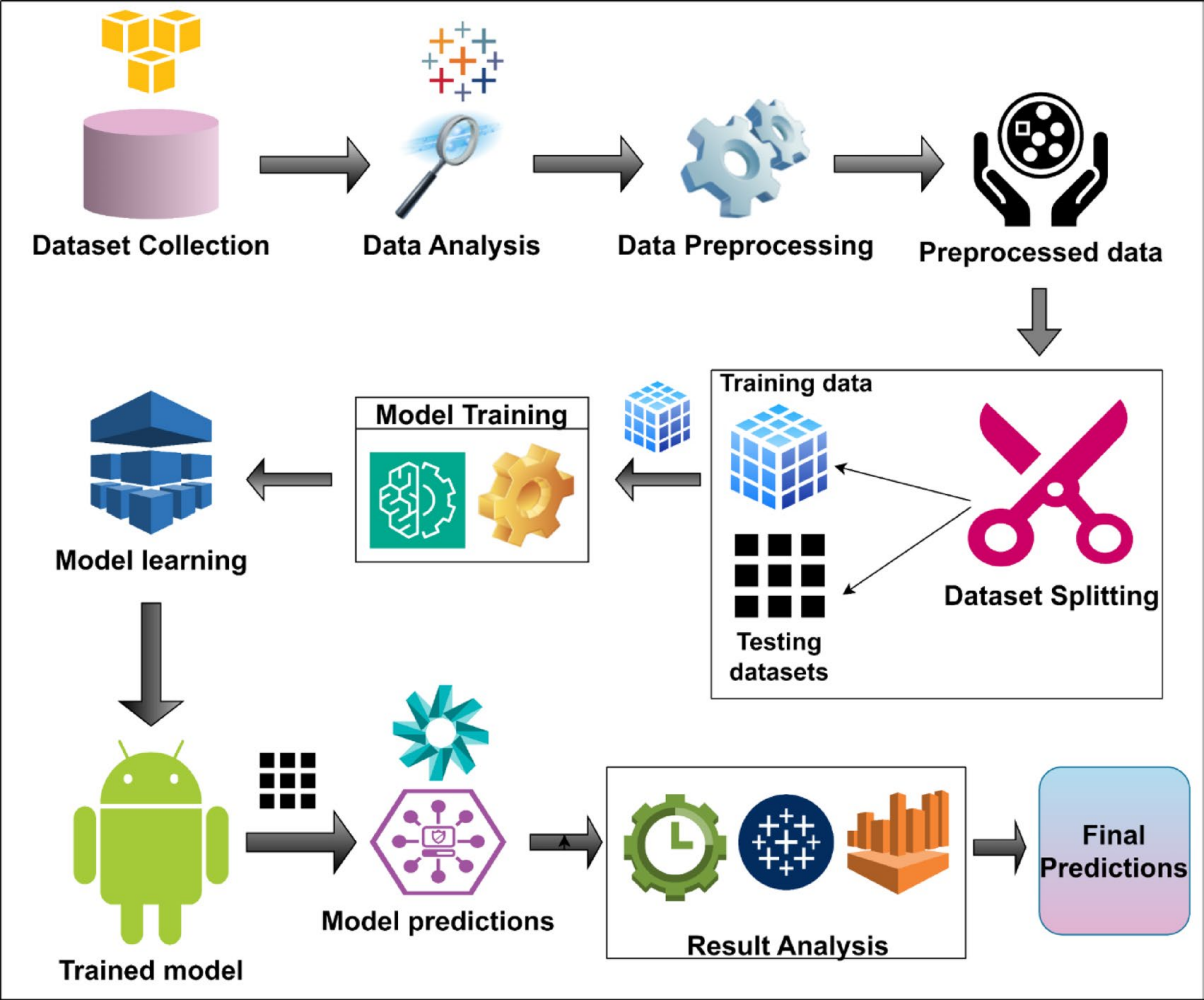
Workflow diagram of Lettuce yield prediction by UniTriRob model.

The workflow diagram in Fig. 3 highlights the different processes that were involved in the Prediction of lettuce crop yield by the UniTriRob regression model. Each phase, starting from dataset collection to final crop yield predictions has been explained in detail as sub-sections.

**Fig. 4:**
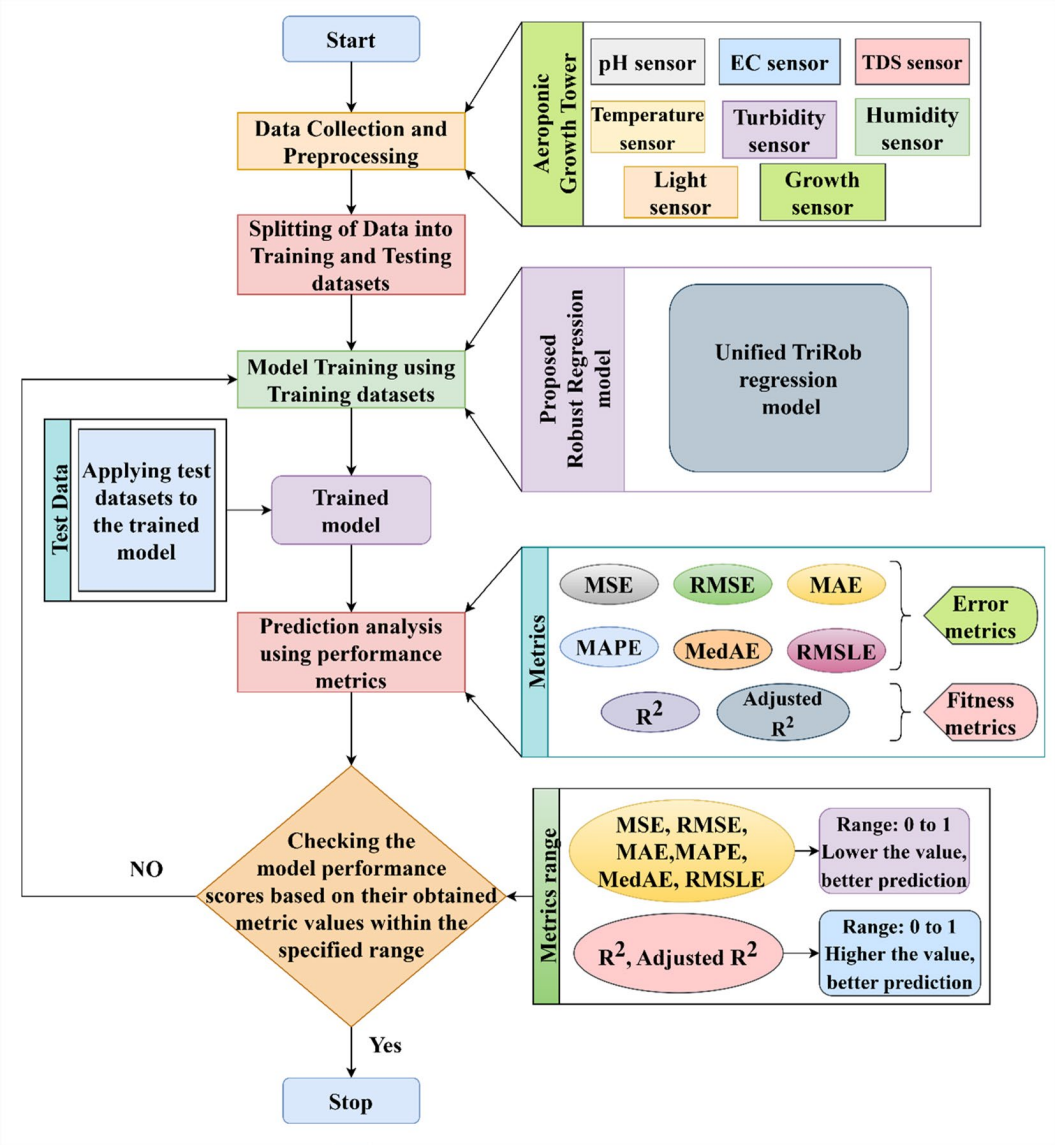
Various phases of the Lettuce Yield prediction system.

The lettuce crop yield is predicted by the robust regression model in the sequence of steps as shown in Fig. 4. The first step is to collect the dataset from the aeroponic tower followed by data cleaning. The dataset is divided into training and testing data. The model is evaluated using the test dataset and trained using the training data. Its performance is assessed using a variety of performance measures. Once the model evaluation had been performed, the analysis of the results was carried out whether the metrics fell within the range; if the metrics fell within the range, then the model accurately predicted the lettuce yield, else the process had to be repeated from model training until the expected results were achieved by the regression model.

#### Dataset collection and pre-processing

The lettuce yield prediction dataset, derived from a 12-month controlled aeroponic farming trial, comprises high-resolution temporal data collected via IoT sensors at 15-min intervals. This multivariate time-series dataset integrates eight critical growth parameters: hydroponic variables (pH, EC, turbidity, TDS), ambient conditions (humidity, temperature, PAR light intensity), and phenotypic metrics (canopy growth rate, final yield [g/plant]). Structured as a 12-dimensional matrix (50,176 rows and 9 columns), the dataset captures three full lettuce growth cycles under varying nutrient regimes. The UniTriRob regression model was trained on this temporal framework to exploit sequential dependencies (e.g., pH drift over time) while mitigating sensor noise, a common challenge in IoT-driven vertical farms. By retaining raw temporal correlations, UniTriRob achieved a lower RMSE in yield prediction compared to non-temporal regression baselines, validating its robustness against heteroscedastic errors endemic to long-duration aeroponic trials. To perform the regression analysis, dataset acquisition is one of the necessary steps where different IoT sensors are deployed to gather these input parameters. Sample lettuce dataset is shown in Table 1.

**Table 1:** Sample lettuce crop dataset.

pH	TDS	Temperature (°C)	EC	Turbidity	Humidity	Light	Growth	Yield
6	714.0	27.0	1.72	204.0	75.867	9.7	22.0	60.0
7	632.0	26.0	0.28	205.0	75.867	9.7	23.0	60.0
7	631.0	27.0	1.34	206.0	75.867	9.7	24.0	65.0
8	663.0	26.0	1.84	207.0	257.476	9.7	25.0	67.0
8	674.0	27.0	6.55	208.0	269.023	9.7	26.0	69.0
8	660.0	26.0	1.43	209.0	261.476	9.7	27.0	70.0
8	677.0	27.0	6.88	210.0	293.476	9.7	28.0	71.0

**Fig. 5:**
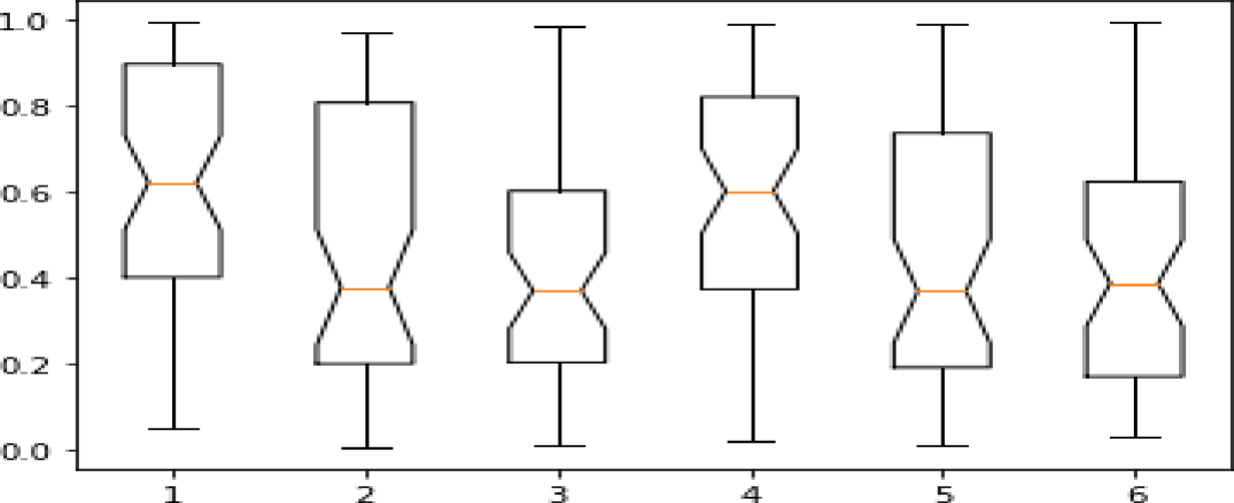
Boxplot visualization for a single growth parameter.

**Fig. 6:**
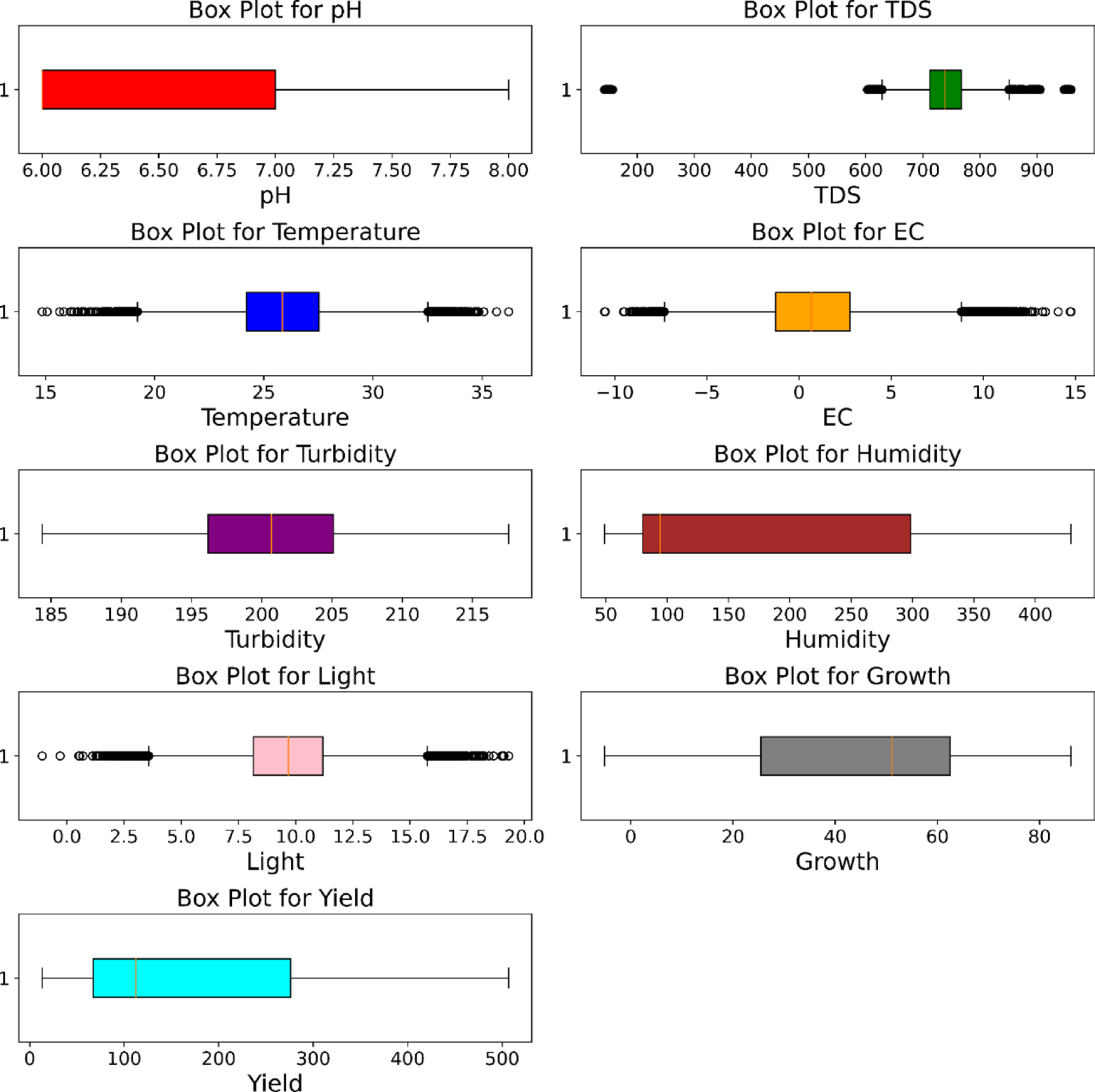
Boxplot visualization in the presence of outliers.

Initially, the UniTriRob model was trained on the original dataset without removing the outliers. Since robust regression techniques were designed to reduce the influence of extreme values rather than depend on prior outlier removal, all type of raw observations were being retained. But, in order to improve the performance of the model, data preprocessing is carried out which mainly focuses on the removal of outliers, and heteroskedasticity was handled by the UniTriRob regression technique. Totally sixty days dataset was collected from the aeroponics tower which consists of 50,176 rows and 9 columns. The entire dataset was visualized using the boxplot representation in Fig. 5 (represents boxplot for a single lettuce growth parameter) and Fig. 6 were demonstrated in the presence of outliers for each lettuce growth parameter with different colors.

**Fig. 7:**
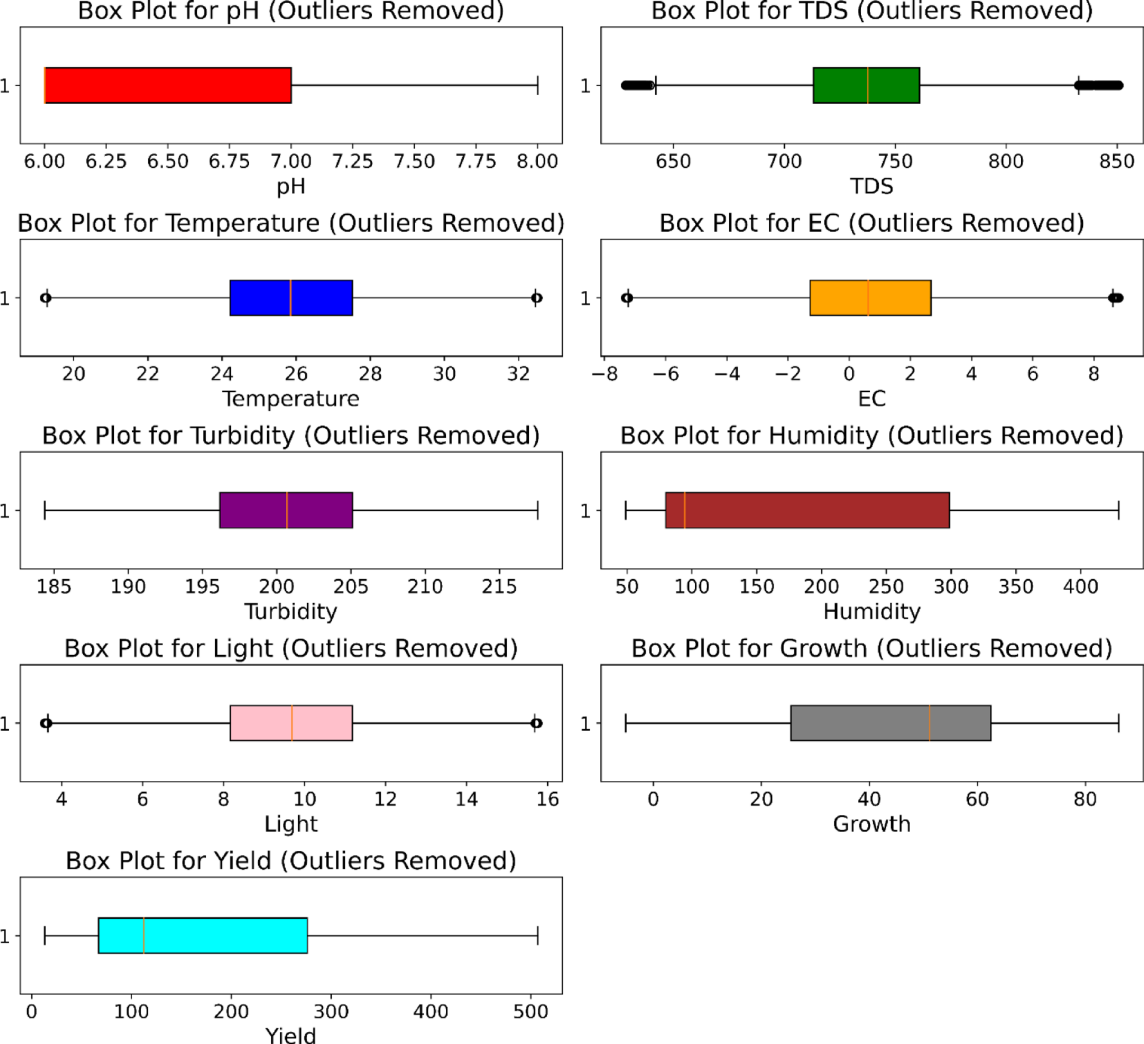
Boxplot visualization after removal of outliers.

Figure 7 showcases the dataset illustration without outliers with the help of a boxplot. Here, all the unnecessary features that were considered as outliers have been removed from the dataset. This helps to minimize the prediction error and increase the performance of the model for better yield prediction.

The raw dataset comprises 50,176 sensor readings with 9 variables, collected at 15-min intervals from the aeroponic tower system. For regression analysis, these raw time-series records were aggregated into 1200 + representative samples corresponding to distinct lettuce growth intervals. The aggregated dataset was then split into 80% for training and 20% for testing to develop and evaluate the proposed UniTriRob model.

#### Dataset visualization

Rather than a theoretical understanding of dataset distribution, the graphical representation can help one to easily fetch the information on how the dataset is distributed among themselves. Here are some of the data visualization techniques for representing the lettuce crop dataset in the form of pictures.

**Fig. 8:**
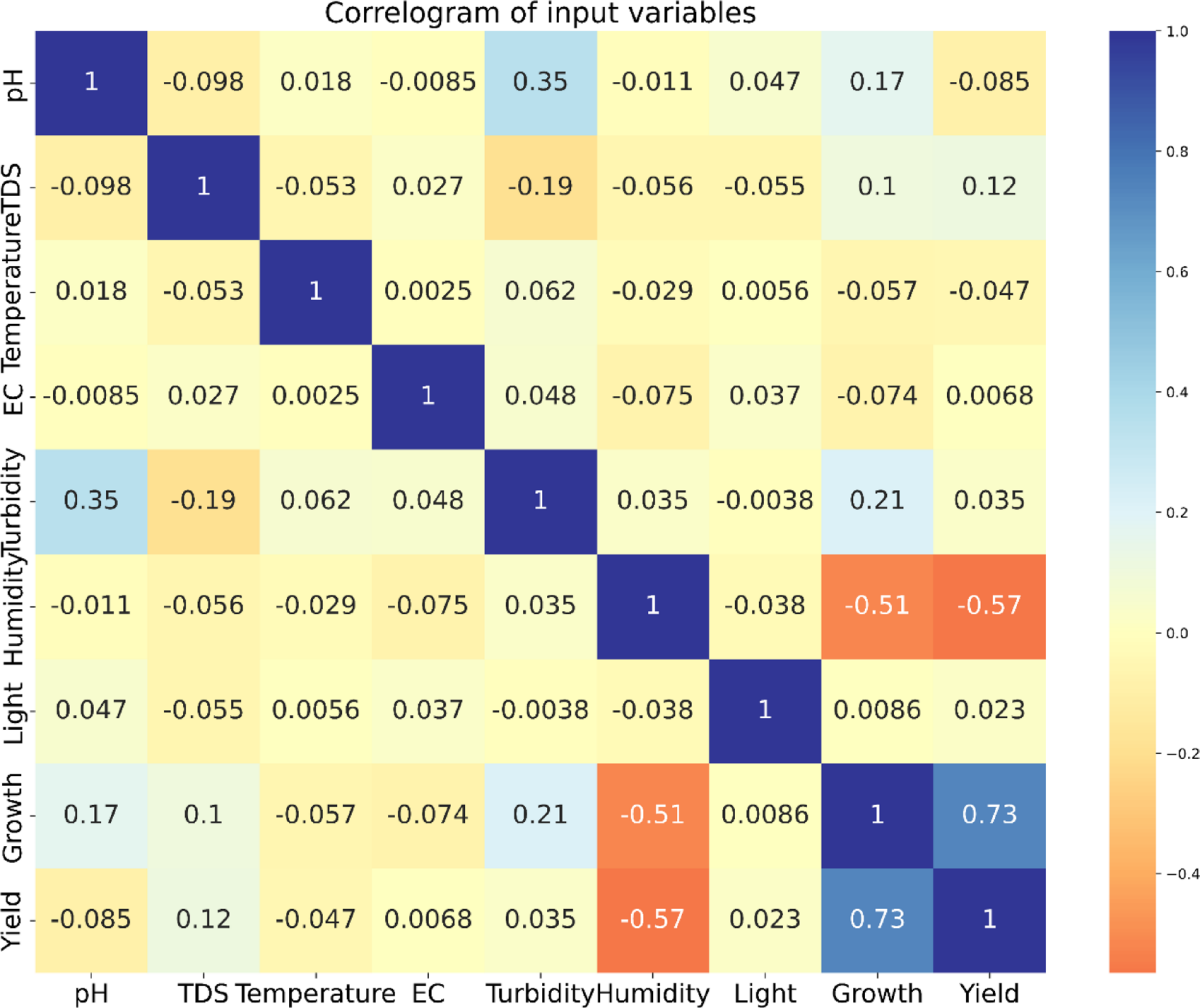
Correlogram of input parameters.

**Fig. 9:**
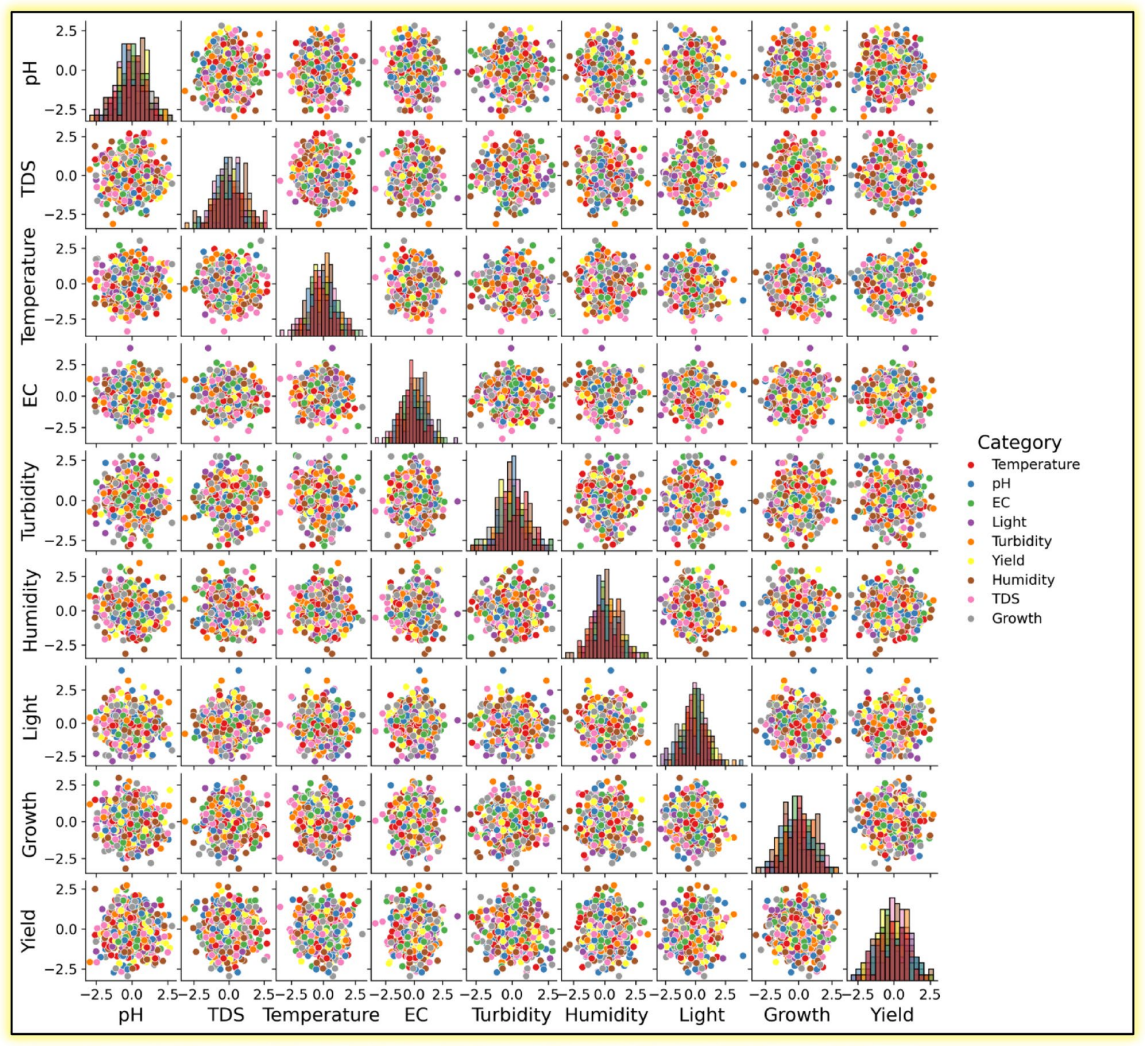
Pairs plot representation of input parameters.

**Fig. 10:**
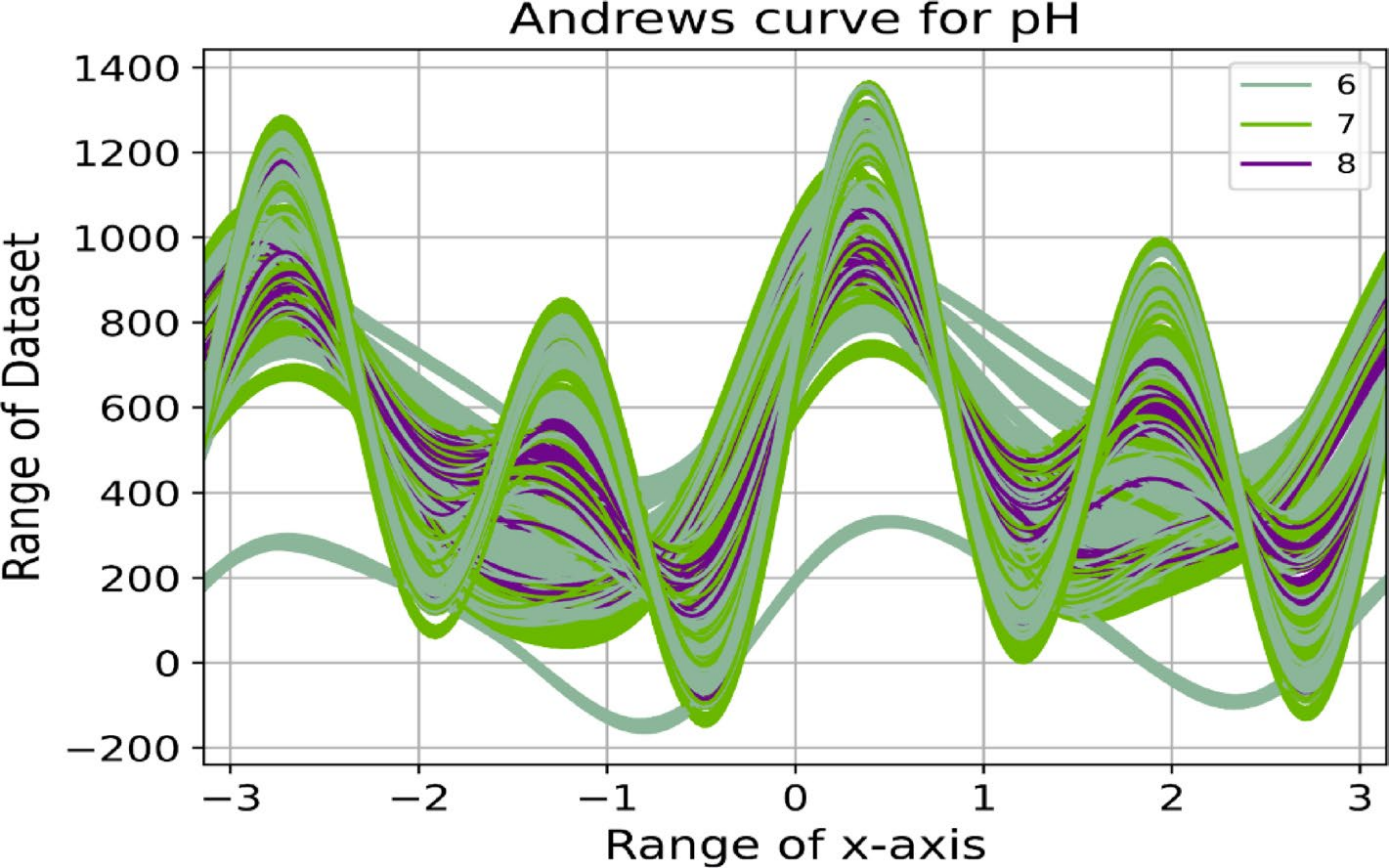
Andrews curve of input parameter pH.

**Fig. 11:**
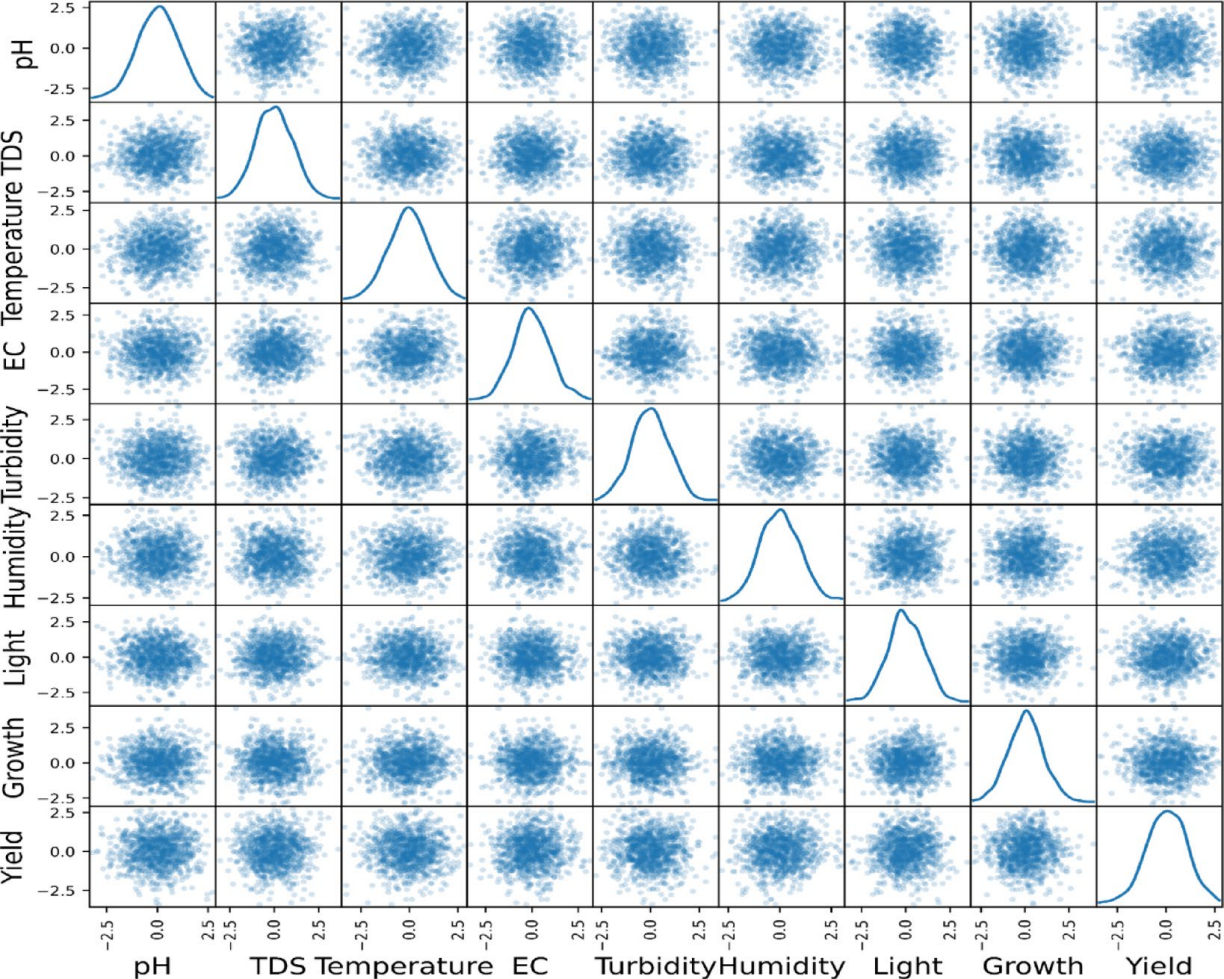
Scatter matrix plot for input parameters.

**Fig. 12:**
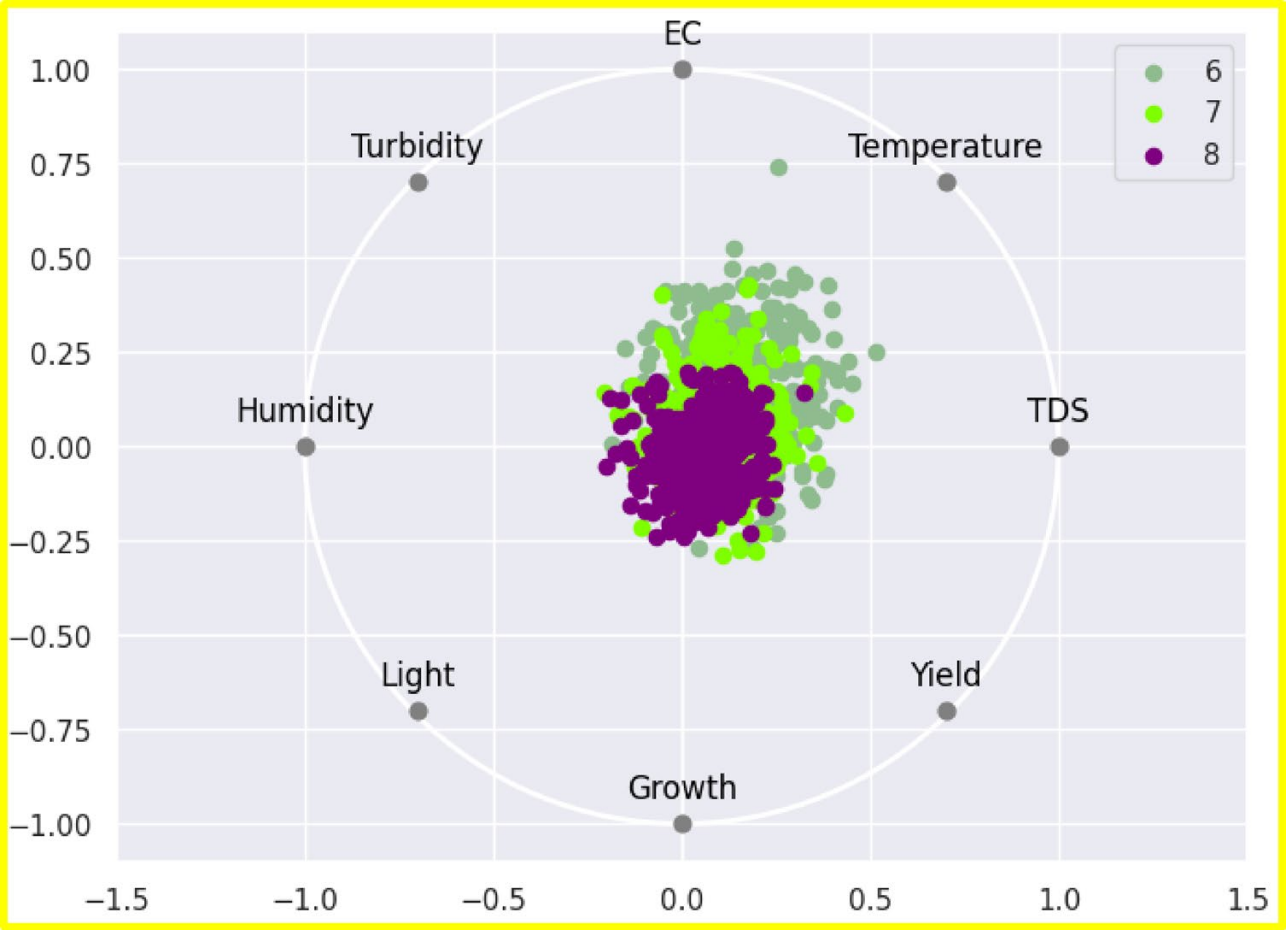
RadViz plot representation of input parameters concerning pH.

**Fig. 13:**
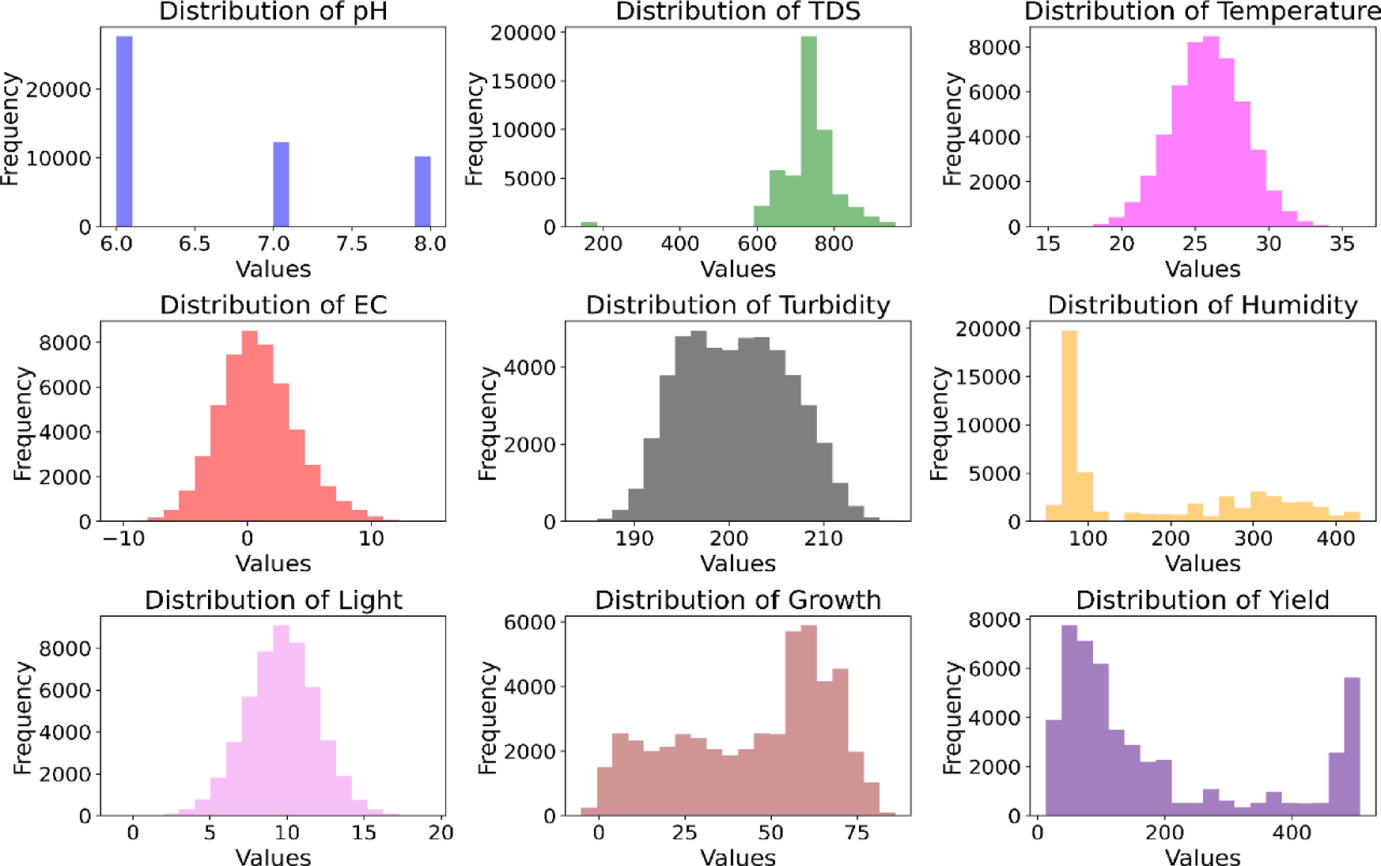
Multiple histograms of the input variables.

**Fig. 14:**
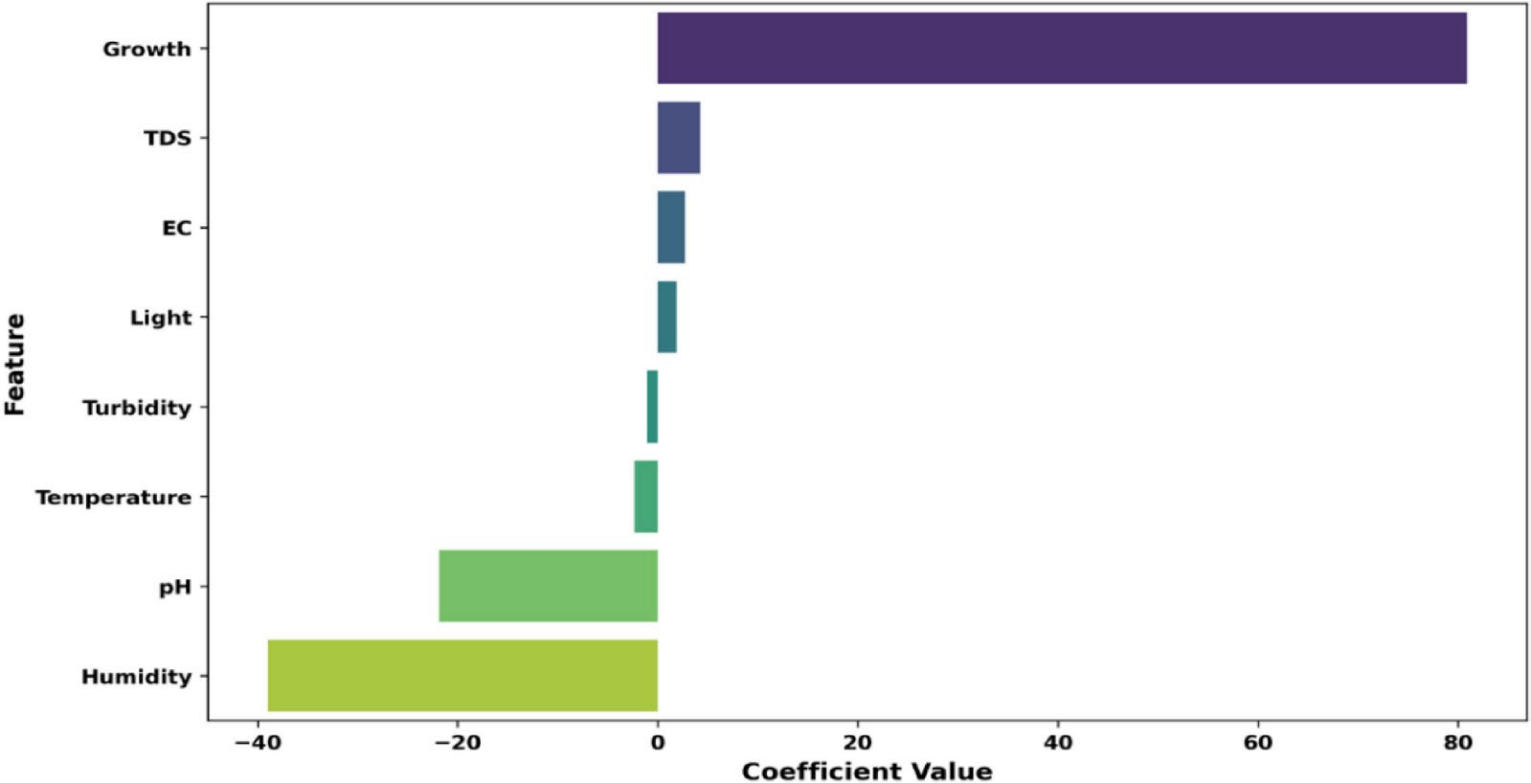
Coefficient bar plot of feature importance.

- Correlogram of the input variables: In data analysis, a correlogram is the visualization chart of correlation statistics that is used for analyzing time-series data. For the lettuce crop yield prediction, the correlogram is displayed in Fig. 8.
- Pairs plots: It is used to represent the values of two different numeric variables which are used to observe the relationship between the variables. For the lettuce crop dataset, the pairs-scatter plot is shown in Fig. 9.
- Andrews curve: It is used to visualize the underlying structure of multidimensional data. It maps all the features from a single observation or rows of data onto a function. The Andrews curve for the input parameter pH is shown in Fig. 10.
- Scatter matrix plot: It is used to explore the relationship between the multiple variables in the presence of missing data in the dataset. It is created by scatter plots, plotting each variable against every other variable. This visualization in Fig. 11 helps to identify the patterns and relationships in the data and any missing data points that need to be attributed.
- RadViz plot: Radial Visualization or the RadViz plot is a multivariate dataset visualization technique that is used to analyze the relationship of one particular input variable with the other variables. Here, each parameter in the dataset is represented by a unique axis emerging from the center of the plot which forms a radial pattern in a 2D plane as represented in Fig. 12.
- Multiple histograms: Here, rather than showcasing all the input parameters within the single image, multiple histograms were utilized to display the input parameters as a separate histogram within the same frame is shown in Fig. 13.
- Feature Importance Visualization: A coefficient bar plot represented in Fig. 14a, which is helpful when considering a large number of input features for predictions, has been used to illustrate the significance of features for the intended research. Based on the positive (bars > 0) and negative (bars < 0) impacts, as shown in Fig. 14a, we can identify and understand the most important variables influencing the growth of the lettuce crop from this plot.

#### Dataset splitting

Data splitting is important phase of algorithms for machine learning, where the dataset is divided into an 80:20 ratios, with 80% used for training and 20% for testing, affecting the predictive model’s performance.

##### Hyperparameter configuration

It is necessary to setup the hyperparameters for ensuring the effective machine learning regression predictions. They are represented as follows: learning rate- 0.05, subsample size- 0.6*n, huber threshold- 1.2, outlier penalty coefficient- 0.65. These essential parameters clearly explain how the regression model could be trained for performing the accurate lettuce yield forecasting.

##### Model training and testing

The training phase, which is the most crucial part of the machine learning model, starts once the data splitting procedure is finished. The model was effectively trained using the training dataset. The trained model is the result of the training step. The testing stage follows. After the test dataset was fed into the training model, the model generated results based on the lettuce time-series data that was supplied in relation to the trained data.

## Results and discussions

This section explains the implementation steps carried out on the lettuce crop yield prediction process. The implementation is done on the Windows 10 operating system; Anaconda Navigator (offline execution) and Google Colab (online execution) were used as the programming tools; Python is used as the programming language.

### Performance evaluation

Once the model training and testing are done, the performance evaluation of the models has to be carried out. Here, to measure the performance of the robust regression models, different performance metrics were used. The detailed descriptions of those evaluation metrics are given below with their mathematical equations respectively. Multiple performance metrics are reported to provide a comprehensive evaluation of the proposed UniTriRob model under different error characteristics (absolute, squared, logarithmic, and percentage-based), which is particularly important for robust regression in the presence of outliers and heteroscedasticity in aeroponic sensor data.

#### Mean squared error

The Mean Squared Error (MSE) is a statistical measure of the average squared difference between the actual and expected values of lettuce output. The squared residuals between the actual and expected values are averaged to determine it.

12 $$MSE={\left(\frac{1}{n}\right)}^{*}\sum {(y\_pred-y\_actual)}^{2}$$

where n is the number of observations, y_actual is the actual yield value, and y_pred is the expected yield value for the lettuce. Table 2 showcases the MSE scores of regression models. The proposed model produces the minimum error rates when compared to other traditional regression models.

**Table 2:** MSE analysis.

Model type	Linear	Polynomial	Quadratic	SVR	Huber	RANSAC	Theil-Sen	UniTriRob
MSE values	0.80303	0.83971	0.47411	0.32060	0.79734	1.02001	0.813533	0.22001

#### Root mean squared error

The Root Mean Square Error (RMSE) is a commonly used statistic that quantifies the average deviation between expected and actual lettuce yield numbers. It imposes harsher penalties on major mistakes compared to small ones, resulting in a higher sensitivity to extreme values than the Mean Squared Error (MSE).

13 $$RMSE=\sqrt{(MSE)} (or)\sqrt{{\left(\frac{1}{n}\right)}^{*}\sum {(y\_pred-y\_actual)}^{2}}$$

Table 3 highlights the RMSE scores of different regression models. The proposed model produces the minimum RMSE score of 0.46905 when compared to other traditional regression models.

**Table 3:** RMSE analysis.

Model type	Linear	Polynomial	Quadratic	SVR	Huber	RANSAC	Theil-Sen	UniTriRob
RMSE values	0.89611	0.91635	0.68855	0.56621	0.89	1.01	0.90	0.46905

#### Mean absolute error (MAE)

The average absolute difference between the predicted and actual lettuce yield values is known as the Mean Absolute Error (MAE). The calculation involves averaging the absolute residuals between the actual and anticipated values.

14 $$MAE={\left(\frac{1}{n}\right)}^{*}\sum (y\_pred-y\_actual)$$

Table 4 showcase the MAE scores of the regression models. The proposed model produces the minimum MAE score when compared to other traditional regression models.

**Table 4:** MAE analysis.

Model type	Linear	Polynomial	Quadratic	SVR	Huber	RANSAC	Theil-Sen	UniTriRob
MAE values	0.64189	0.69352	0.49828	0.5908	0.64006	0.79717	0.66921	0.39717

#### Mean absolute percentage error

The average percentage difference between expected and actual values is known as MAPE, and larger deviations are indicated by higher values. In terms of mathematics, it is expressed as

15 $$MAPE=\frac{\left[\frac{\sum (y\_actual-y\_pred)}{\sum (y\_actual)}\right]}{n}$$

Table 5 highlights the MAPE scores of the proposed model which produced the minimum MAPE score of 0.05368 when compared to other regression models.

**Table 5:** MAPE analysis.

Model type	Linear	Polynomial	Quadratic	SVR	Huber	RANSAC	Theil-Sen	UniTriRob
MAPE values	0.37	0.26	0.21	0.15	0.06740	0.08338	0.17	0.05368

#### Median absolute error (MedAE)

The median total discrepancy between the expected and actual value is a reliable indicator of a model’s performance since it is less affected by extreme values compared to metrics that are influenced by the mean.

16 $$MedAE=median(y\_actual-y\_pred)$$

Table 6 showcase the MedAE scores of the regression models. The UniTriRob model produces the MedAE score of 0.44382, which is less than the other traditional regression models.

**Table 6:** MedAE analysis.

Model type	Linear	Polynomial	Quadratic	SVR	Huber	RANSAC	Theil-Sen	UniTriRob
MedAE values	0.57389	0.78653	0.67439	0.53421	0.48161	0.64482	0.48161	0.44382

#### Root mean square logarithmic error

Root Mean Square Logarithmic Error (RMSLE) is a metric that quantifies the average logarithmic difference between the actual and projected values, often used for target variables that have a broad range. The calculation involves taking the natural logarithm of both values and then determining the difference, with a greater penalty for underestimation compared to overestimation.

17 $$RMSLE=\sqrt{{\left(\frac{1}{n}\right)}^{*}\sum [{log{(y}_{actual}+1)- log\left({y}_{pred}+1\right)]}^{2}}$$

Table 7 highlights the RMSLE scores of the proposed model which produced the minimum logarithmic error score of 0.02929 when compared to other regression models.

**Table 7:** RMSLE analysis.

Model type	Linear	Polynomial	Quadratic	SVR	Huber	RANSAC	Theil-Sen	UniTriRob
RMSLE values	0.08431	0.03496	0.06742	0.09847	0.07113	0.09028	0.07114	0.02929

#### R-squared metric (coefficient of determination)

R^2^ ranges from 0 to 1, where a value of 1 indicates the perfect fit and a value of 0 represents no relationship between the variables.

18 $$R^{2} = 1 - \left[ {\frac{{\sum \left( {y\_actual - y\_pred} \right)^{2} }}{{\sum \left( {y\_actual - y\_mean} \right)^{2} }}} \right]$$

where y_mean is the mean value of the lettuce yield.

Table 8 represents the R-squared metrics of the regression models. The UniTriRob model produced the R-squared value of 97.8386, which is higher than the other traditional regression models that describe the better fitting of the model to the lettuce dataset.

**Table 8:** R-squared metrics analysis.

Model type	Linear	Polynomial	Quadratic	SVR	Huber	RANSAC	Theil-Sen	UniTriRob
R^2^	20.893	54.528	41.011	80.893	87.982	88.538	53.501	97.8386

#### Adjusted R-squared metric

Adjusted R-squared metric adjusts the R-squared value by penalizing the number of predictor variables in the model. It takes into account the model complexity and prevents the overfitting problem. The adjusted R^2^ value may be more than or equal to the R^2^ value when additional predictor variables contribute significantly to the model’s predictive power.

19 $$Adjusted R^{2} = 1 - \left[ {\frac{{(1 - R^{2} )\left( {n - 1} \right)}}{{\left( {n - p - 1} \right)}}} \right]$$

where, n is the number of observations and *p* is the number of independent variables.

Table 9 highlights the adjusted R-squared metrics which is the improvised score of the R-squared metrics. Higher the metric values, better the predictive performance by the model.

**Table 9:** Adjusted R-squared analysis.

Model type	Linear	Polynomial	Quadratic	SVR	Huber	RANSAC	Theil-Sen	UniTriRob
Adjusted R^2^	28.548	10.729	54.385	65.483	88.542	94.035	90.175	97.865

### Performance comparison of the proposed model with other regression models

**Fig. 15:**
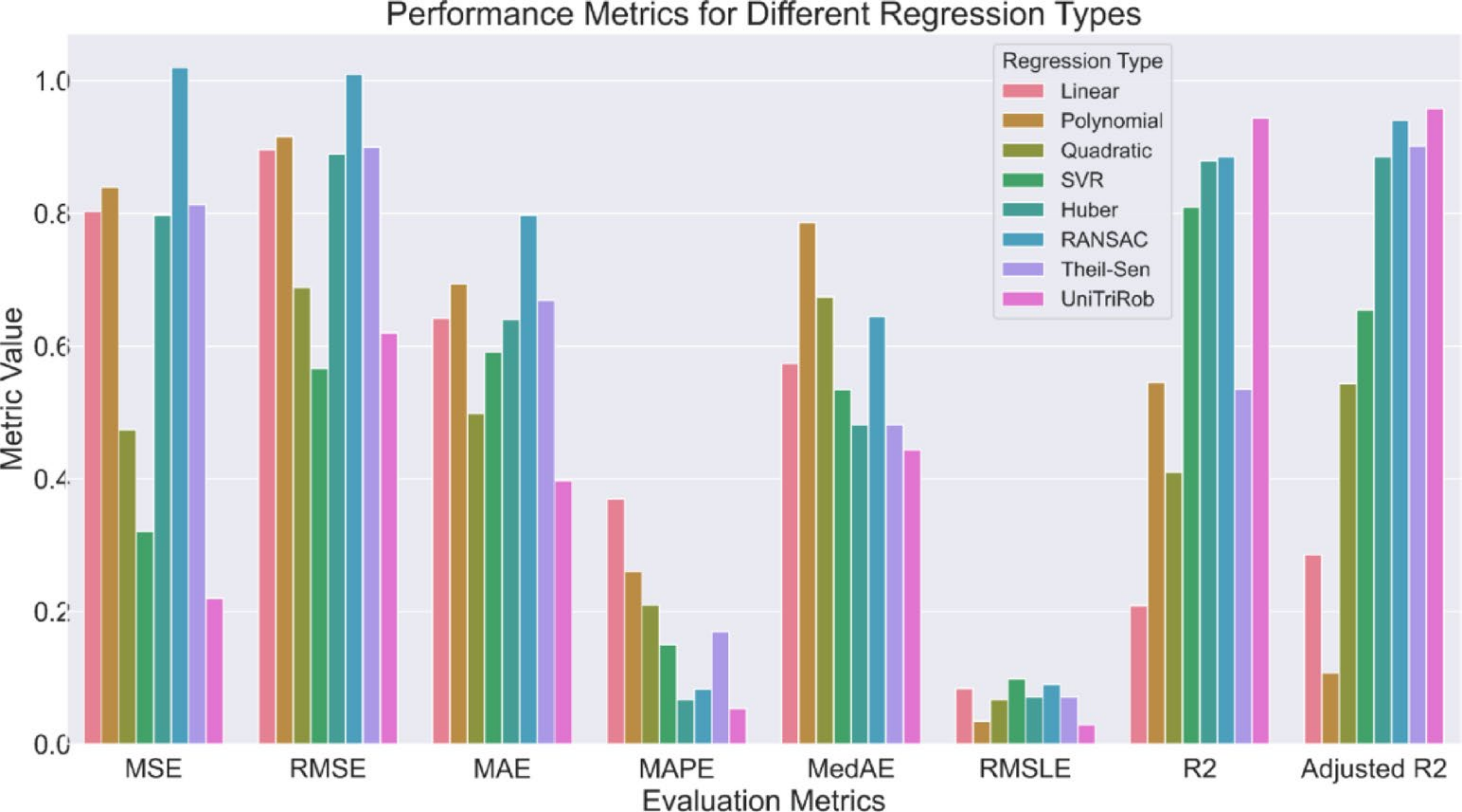
Comparison of the predictive metrics across various models.

**Fig. 16:**
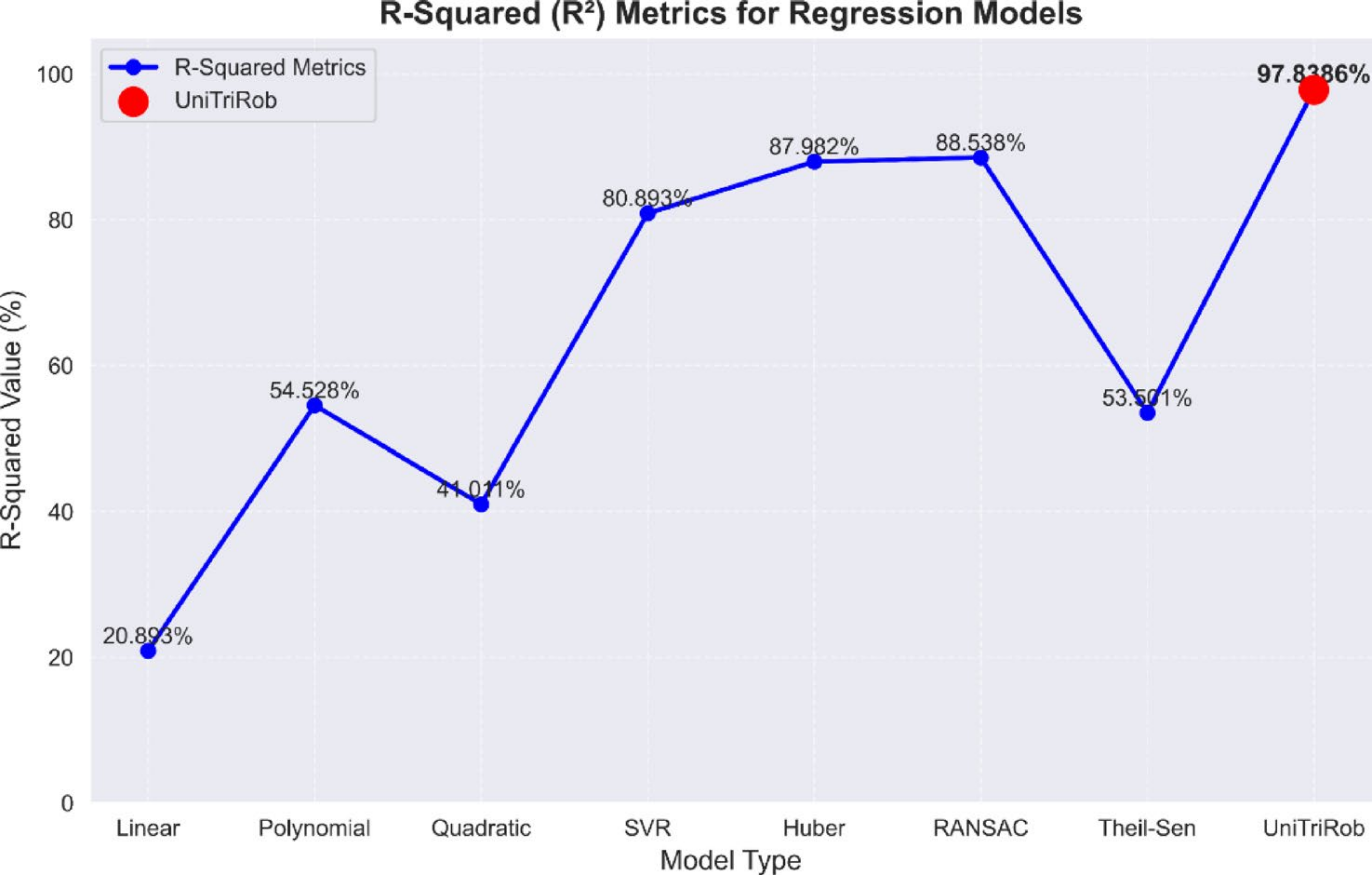
Performance comparison of UniTriRob model with other regression models.

The final regression performance of the model is highlighted in Table 10 based on the obtained performance metrics. The UniTriRob model outperforms other regression models in predicting outcomes with high model performance. As shown in Table 10, Figs. 15 and 16, its low MSE and RMSE result in predictions that closely resemble actual outcomes. The MAE and MedAE show precision, with a small difference between anticipated and actual values. The model’s MAPE of 0.05368 ensures low percentage errors, ensuring reliable predictions across different data points. The RMSLE of 0.02929 indicates the model’s ability to make accurate predictions over a broad range of values. The error metrics MSE, RMSE, MAE, MAPE, MedAE and RMSLE were measured in terms of lettuce yield which is in grams. In case of R2 and Adjusted R2 metrics, they are measured in terms of percentage.

**Table 10:** Comparison of the predictive metrics across various models.

S. No	Model type	MSE (in grams)	RMSE (in grams)	MAE (in grams)	MAPE (in grams)	MedAE (in grams)	RMSLE	R^2^ (in %)	Adjusted R^2^ (in %)
1	Linear	0.80303	0.89	0.64189	0.37	0.57389	0.08431	20.893	28.548
2	Polynomial	0.83971	0.91	0.69352	0.26	0.78653	0.03496	54.528	10.729
3	Quadratic	0.47411	0.68	0.49828	0.21	0.67439	0.06742	41.011	54.385
4	SVR	0.32060	0.56	0.5908	0.15	0.53421	0.09847	80.893	65.483
5	Huber	0.79734	0.89	0.64006	0.067	0.48161	0.07113	87.982	88.542
6	RANSAC	1.02001	1.01	0.79717	0.083	0.64482	0.09028	88.538	94.035
7	Theil-Sen	0.813533	0.90	0.66921	0.17	0.48161	0.07114	53.501	90.175
8	UniTriRob	0.22001	0.46	0.39717	0.053	0.44382	0.02929	97.8386	97.865

The UniTriRob (Proposed) model shows a high level of fit, with an R-squared (R^2^) value of 0.9783 and an Adjusted R-squared (Adjusted R^2^) value of 0.9786. This suggests a substantial amount of the variation in the dependent variable while considering model complexity. The UniTriRob (Proposed) model is emphasized as a precise and dependable regression model, ideal for predictive modeling in several fields.

### Validation loss and training loss plots

The evaluation process serves a critical purpose by indicating that the developed model not only fits the training data but also effectively generalizes to new data. Two distinct performance metrics, namely training loss and validation loss, play a crucial role in demonstrating the efficacy of machine learning models during the training phase, particularly in regression problems. These loss functions can be dissected into two categories:

*Training loss* During training, the model aims to minimize the training loss function. A lower training loss signifies better model performance. In the case of our proposed ensemble model, the loss functions yield negative values, suggesting that the model outperforms the baseline or initial state. The significance of these more negative values lies in the model’s superior ability to fit the training data compared to a trivial model. The magnitude of the reduced values quantifies how much better the model is relative to the baseline.

*Validation loss* The validation loss holds greater importance than the training loss as it provides insights into the model’s performance on new, unseen data. What sets the validation loss apart is the use of a separate dataset that was not employed during the training phase for evaluation. This validation phase assesses the proposed model’s predictive capacity, specifically for crop yield prediction.

*Activation functions and optimizers* Activation functions, such as ReLU, Leaky ReLU, softmax, sigmoid, and tanH, are essential in introducing non-linearity to models, allowing them to capture complex data relationships and converge on intricate patterns, making them widely adopted due to their simplicity and effectiveness. On the other hand, Optimizers are optimization algorithms used to improve model performance, such as SGD, Adam, Root Mean Square Propagation (RMSProp), and Adagrad, which are crucial in real-time applications.

**Fig. 17:**
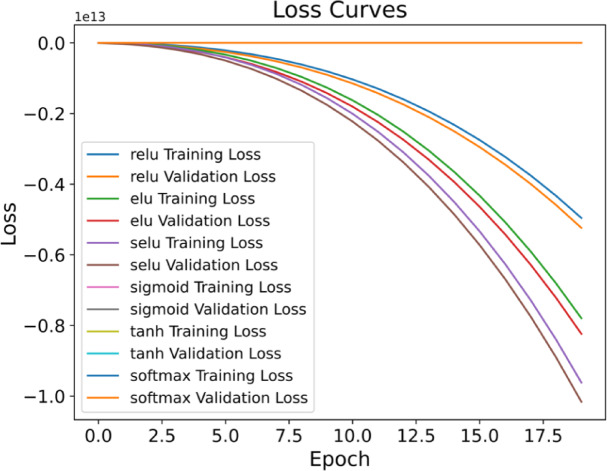
Training loss and Validation loss representation for the UniTriRob regression model.

*Interpretations from the loss plots* As discussed in the earlier sub-section, the validation loss and the training loss are the important loss functions to determine the effectiveness of the developed machine learning model specifically for performing the regression problems. Here, the efficiency of the proposed ensemble model was interpreted for lettuce yield prediction based on the provided crop growth dataset. For each plot, 20 total number of epochs were used. Different activation functions with the same optimizer called “Adam” were highly utilized. The graphical representation of the loss metrics is represented below in the Fig. 17.

### Prediction graphs

**Fig. 18:**
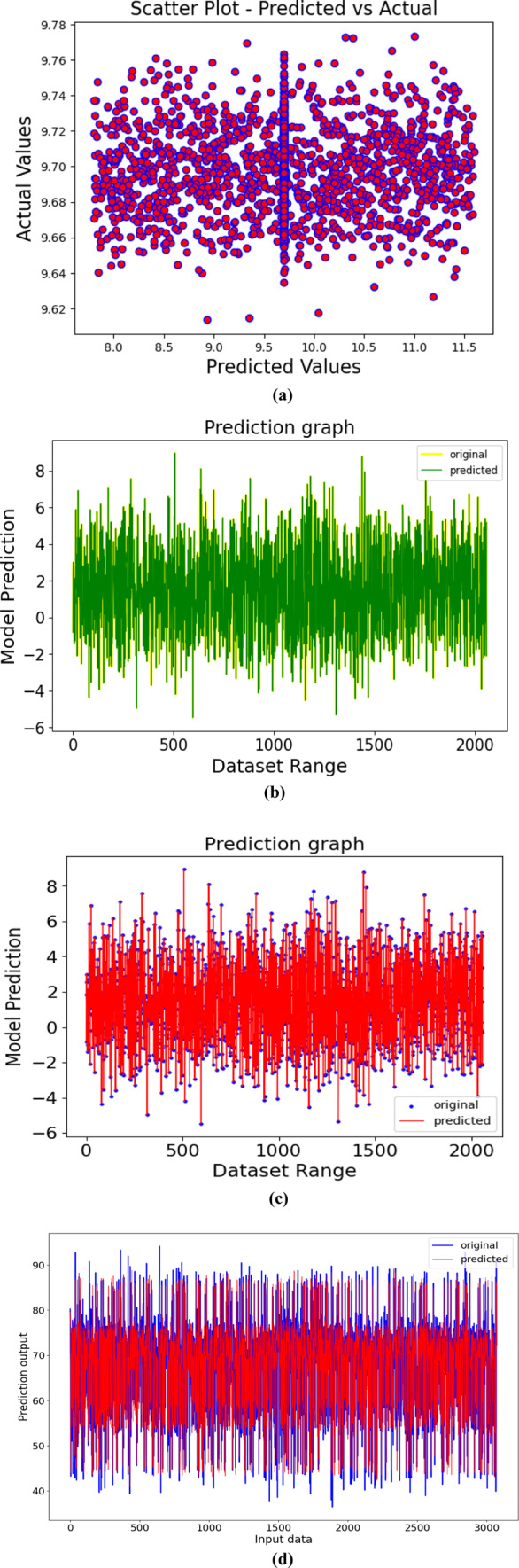
(**a**)–(**d**) Represents the different types of Prediction Graphs produced by the UniTriRob regression model.

While using the robust regression models for prediction, one can visualize the prediction results through the graph plotting to gain the information about how well the model works and how the predictors and the predicted variable relate to one another. In this case, scatter plot is used for comparing the actual values and the predicted variable which is lettuce crop yield against the predicted values obtained from the robust regression models. Here, each point on the plot represents an observation, where X-axis represents the predicted values (yield) and Y-axis represents the actual values (input variables). A well-performing regression model would show the tight clusters of points close to the line (y = x), indicating a strong relationship between the predicted values and the actual values. Hence, the proposed model could be utilized for forecasting the yield of the lettuce crop grown in the aeroponic system. Different types of prediction graphs produced by the UniTriRob regression model are shown in Fig. 18a–d.

### Time complexity analysis of the robust regression models with the UniTriRob model

Assessing the computational efficiency of forecasting aeroponic lettuce production relies heavily on analyzing the temporal complexity of resilient regression models. By examining the execution times of the robust regression models- Huber, RANSAC, Theil-Sen and Unified TriRob regression model one can gain deeper insights into their time complexities and determine which model offers the most efficient computational performance.

*Huber Regression* Huber regression is an iterative algorithm that minimizes a combination of squared errors and absolute errors. The time complexity of Huber regression is typically,

20 $$O(k*n*d)$$

where, k is the number of iterations, n is the number of data points and d is the number of features.

The time complexity increases linearly with the number of iterations, data points and features. Thus, the computational cost of Huber regression grows proportionally to the size of the dataset and the number of iterations required for convergence. For the implemented aeroponic lettuce crop yield prediction system, the Huber model’s time complexity was 3.0254298 s.

*RANSAC regression *RANSAC is an iterative algorithm that fits the models to random subsets of the data, aiming to identify the inliers and estimate the optimal model parameters. The time complexity of RANSAC regression is typically,

21 $$O(k*s*t)$$

where k is the number of iterations, s is the sample size, t is the number of trials.

The time complexity depends on the number of iterations and trials needed to obtain a satisfactory model. Consequently, the computational complexity of RANSAC regression grows with the number of iterations and trials, as well as the sample size. For the implemented aeroponic lettuce crop yield prediction system, the RANSAC model’s time complexity was 2.2499008 s.

*Theil-Sen regression* Theil-Sen regression is a non-parametric method that estimates the slopes of the median of all pairs of data points. The time complexity of Theil-Sen regression is typically,

22 $$O({n}^{2})$$

where *n* is the number of data points.

As a result, the time complexity of Theil-Sen regression increases quadratically with the number of data points. It should be noted that for layer datasets, the computational cost of Theil-Sen regression can become prohibitively high due to its quadratic time complexity. For the implemented aeroponic lettuce crop yield prediction system, the Theil-Sen model’s time complexity was 13.4485617 s.

*UniTriRob model* The time complexity of the proposed model could be found analyzing the computational costs of its main operations such as computing robust loss and matrix functions for determining the coefficients of the features. The mathematical formula for finding the time complexity is given by,

23 $$O(k.m.\left(n+p\right)+k.n.{p}^{2})$$

where, $n$ is the number of observations which is nothing but the size of the dataset, $p$ is the features that is dimensionality of the input data, $k$ is the number of iterations required for convergence and $m$ is the data points used in each iteration for robust estimation.

Equation (23) is broken into two terms as follows,

- $O(k.m.\left(n+p\right))$ represents the computational cost of the robust regression which represents iteration $k$ times, each time processing of $m$ datapoints and for each point, calculating residuals with $n$-operations and updating weights of additional $p$ operations.
- $O(k.n.{p}^{2})$ accounts for the computation cost of the matrix operations, including matrix multiplication and inversion, which are required for estimating the coefficients.

This time complexity equation provides an estimate of the computational resources required for execution of the UniTriRob model based on the specifications.

#### Comparison of time complexities of Huber, RANSAC, Theil-Sen and UniTriRob model

The measured execution times by the robust regression models were comparatively analyzed in the below Table 11:

**Table 11:** Time complexity analysis of robust regression models.

Regression type	Number of iterations	Time complexity (in seconds)
Huber	10	3.0254298
RANSAC	10	2.2499008
Theil-Sen	10	13.4485617
UniTriRob	10	1.98956472

**Fig. 19:**
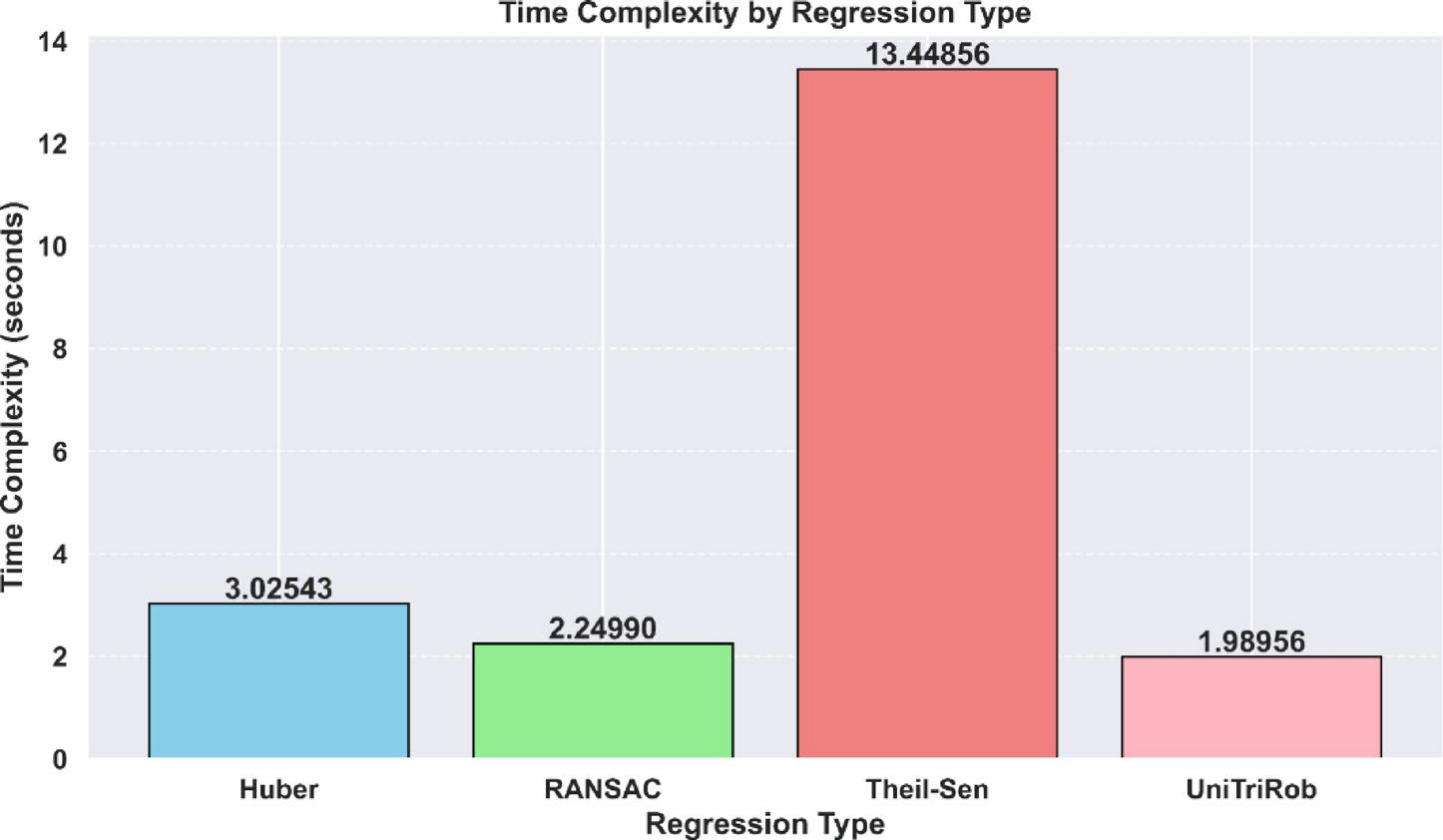
Time complexity analysis of UniTriRob model with other robust regression models.

The proposed UniTriRob model outperforms other robust regression models, as shown in Fig. 19, in terms of computational efficiency for predicting lettuce crop yield in aeroponic systems. Unlike RANSAC, UniTriRob does not rely on random subset selection for inlier identification. Instead, its lower execution time is attributed to adaptive residual weighting and an efficient iterative optimization strategy, which enables faster convergence while maintaining robustness to outliers and heteroscedastic noise.

The proposed regression, in effect, reduces computational overhead, making it preferred for prediction of crop yield since it offers efficient computational performance and is specifically beneficial when handling large datasets or situations where prediction should be performed timely. Nevertheless, their application to large scale datasets of lettuce crop requires special treatment to reasonable time complexity in terms of robust regression models. Algorithms with higher time complexity may result in an increase computation time if the dataset size increases.

To determine precisely a most suitable robust regression model for the prediction of the lettuce crop yield in aeroponics systems, there is a need for a thorough evaluation involving time complexity and the rest of relevant factors. The proposed model turns out to be the most time efficient; however, the factors and requirements concerning its implementation have to be taken into account.

## Conclusion and future scope

Certain crops grown in the misted environment without the use of soil is a unique way of growing plants which is called aeroponics. The detailed description was made on how the robust regression models (UniTriRob) is used to predict the lettuce crop yield in the system aeroponics. Using this model has displayed the ability to increase crop production and harvest optimization. UniTriRob regression model handles outliers and influential observations, gives better and reliable prediction to lettuce crop yield while mitigating the effect of extreme values allowing more stable predictions leading to better decision making for crop management and resource allocation. Furthermore, the proposed model works the iterative fitting process which promises a better estimation of regression parameters of lettuce crop yield prediction. Using it, growers can determine and tackle potential problem areas that may impede crop yield and, by doing so, improve the utilisation of cultivation. The Theil-Sen regression model which utilizes the median of pairwise slopes, gives robust estimates of regression parameters and leads to insights into growth patterns and yield potential in this unique cultivation environment.

Using these robust regression models, growers and researchers can base their decisions about lettuce crop yield on data and identify the factors affecting yield (such as nutrient levels, environmental conditions and growth stage) for improving yield. This accurate crop yield forecasting enables operators to adjust nutrient concentrations or spray intervals to counteract predicted declines in yield due to nutrient imbalances. Also, this proactive approach helps farmers schedule harvesting and market supply chains in advance, reducing waste and enhancing profitability. However, they need further fine tuning and validation in the framework of the aeroponic system. Further, the influential factors such as input system and plant types will be explored, field experiments will be conducted and more comprehensive datasets can be collected, to drive the continuous improvement of predictive performance and the development of custom fitted models for a particular setup of aeroponics system. It should be noted that the findings reported in this study are limited to lettuce cultivated under a controlled aeroponic setup, and no claims of cross-crop or cross-system generalization are made in the present work. While the proposed model demonstrated high efficacy for lettuce yield prediction, its extension to a wider range of leafy green and fruiting crops will be investigated as part of future work. The reported performance metrics represent average values obtained from multiple independent runs of the UniTriRob model; however, statistical variability measures such as standard deviation or confidence intervals were not explicitly included, which is acknowledged as a limitation of the present study.

## Data Availability

The dataset used in this study was generated from a controlled aeroponic experimental setup. Due to the absence of a public repository at the time of publication, the data are available from the corresponding author upon reasonable request. Also, the authors will consider depositing the dataset in a public repository in future research work to enhance reproducibility and ease to access. Source code: The implementation details of the proposed regression model were described in the manuscript, and the source code would be provided by the corresponding author upon reasonable request. Ethical statement: This research does not involve human participants or animal’s data. The data used in the research work were collected from an IoT-enabled agricultural system as sample and further synthesized using the augmentation technique; therefore, ethical approval was not required.
